# Supplementary material for: Estimation of the lost productivity to the GDP and the national cost of correcting visual impairment from refractive error in Kenya
Source: PLoS One. 2024 Mar 25;19(3):e0300799. doi: 10.1371/journal.pone.0300799 (PMC10962815; doi:10.1371/journal.pone.0300799)
Supplement: S1 Data — (PDF) [file pone.0300799.s001.pdf]

Supplementary

Lost productivity to the GDP

Currently in Kenya, approximately 3.5 million people are estimated to suffer from RE [1]. The lost productivity to the GDP estimates was based on a study by Eckert and colleagues [2] in which MSVI results in a range of reduced productivity with 30%-55% not working while 45%-70% have their earning reduced by 35%. During the estimation, the population aged 16-60 years with VI was included for the estimations with the assumption that the population below 16 years and those above 60 years with URE yield no economic productivity to a country's GDP as proposed by Smith and colleagues [3]. The minimum wage of US$ 101.505 for Kenya was used in the estimation of the lost productivity to the GDP as a result of RE in Kenya. The annual MW for the 45%-70% of the individuals with URE who are working was computed. Thereafter, the reduced earnings for the visually impaired individuals who are working in Kenya was estimated using a factor of 0.35 as proposed by [2]. The productivity loss was computed as a product of (0.45 of the affected population * 0.35 of reduced earnings) and (0.7 of the affected population * 0.35 of reduced earnings) Eckert and colleagues [2].

Productivity benefit of addressing refractive error

In Kenya, approximately 51% of the visually impaired individuals constitute the working age group [4] with an employment rate (ER) of 16% [5]. In the absence of VI, those treated would be employed at the same rate as the individuals with good vision [6]. In Kenya, the national ER is estimated at 38.5% for the population aged 16-60 years with a MW of US$ 101.505 monthly [5]. An assumption was made that if individuals with VI benefits from spectacles and low vision devices, then the ER would potentially be scaled to the national average of 38.5% [5]. The productivity benefit was computed in three perspectives. Firstly, the productivity benefit for the 51% of the individuals who are visually impaired and have attained the employment age was computed as a product of the annual MW and the population [7]. The unadjusted productivity benefit was adjusted using the Labour Force Participation Rate (LFPR) which is the estimate of the economy's active workforce [8]. In Kenya, the LFPR is approximately 0.739 [9]. Secondly, the current productivity benefit accrued from the 16% of the visually impaired in Kenya was calculated. Finally, a productivity benefit was computed with an assumption that the ER which is the measure of the extent to which available labour resources are being utilized for visually impaired is scaled to the national average of 38.5% [8].

Productivity loss of the caregivers

Out of the 3.5 million Kenyans with MSVI, it is estimated that approximately 7.5% have severe VI [10]. Therefore, it was assumed that there is one caregiver for every individual with severe VI. The number of required caregivers was estimated based on the number of individuals with

severe VI in Kenya. Based on a study by Fricke and colleagues [11] , it was assumed that every caregiver for a visually impaired individual experiences a productivity loss of 5% on the total average yearly productivity. Hence the productivity loss of caregivers was computed as a product of the average annual productivity for each caregiver and the 5% productivity loss due to the care provided. The MW for Kenya which is approximately US$ 101.505/month was used.

National cost required to establish vision centres (VCs), scale functional clinical refractionists and provide spectacles based on the estimated prevalence of URE in Kenya.

The cost required to establish the VCs in Kenya was computed based on estimates from the IAPB of US$ 50,000 [12] and the Ministry of Health of Kenya of US$ 100,000 [13] per vision centre (VC). Currently there are approximately 77 vision centres in Kenya and a 1:100,000 and 1:50,000 vision centre per population is to be adopted then 475 and 951 are required respectively. Functional clinical refractionists were defined as individuals who undertakes refraction 100% of their time [14]. In Kenya, there are approximately 560 functional clinical refractionists. Based on the range of services that ophthalmic workers in Kenya undertakes as per their scope of practice [14], this paper estimated that ophthalmologists may spend roughly 20% of their time doing refraction, ophthalmic clinical officers roughly 30% of their time, optometrists may spend approximately 90% of their time and finally ophthalmic nurses may spend roughly 15% of their time [14]. The number of required functional clinical refractionists from the existing eye care professionals having the potential to conduct refraction in Kenya was calculated based on the estimates by Morjaria and collegues [16]. The deficit of functional clinical refractionists was computed based on the WHO recommendation of one functional clinical refractionist per 100,000 populations [17]. The calculations were based on the ratio of one refractionist per 50,000, 25,000 and 10,000 population. The cost required to train functional clinical refractionists was computed for each category of the eye care professional based on the deficit and the recommendations by the WHO, Ministry of Health and this study recommendations based on telemedicine integration. Currently, the cost of training an eye care professional in Kenya is approximately US$ 1,680 - US$ 20,450 [18].

To estimate the number of individuals with URE who already have corrective devices, the 6% estimates of the number of spectacles wearers in Tanzania was adopted [19]. The estimated number of individual with URE already having corrective devices was excluded from the national cost estimates. The national cost for correcting URE for the 3.5 million Kenyans was computed based on the average spectacles charges from the private sector and the SE sectors. The current spectacle charges from the private sector in Kenya is estimated at an average cost of US$ 75 [20] and that of SE an average of US$ 35 [21].

Cost benefit analysis

Considering that the benefit of addressing URE remains significant and mostly overrides cost invested in addressing URE by a factor of 3.5 times [4], the implication is that if one dollar is invested in addressing URE then a possible return of US$ 3.56 will be accrued. Hence the cost benefit analysis was computed as a product of the cumulative national cost required to address URE and the factor of 3.5 times was used. For each US$1 invested in the efforts to eliminate VI in Kenya, a return of US$3.56 could potentially be realized [4].

References

1. Muma S, Naidoo KS, Hansraj R. Estimation of the Prevalence of Refractive Error in Kenya : A Systematic Review and Meta-Analysis. Optom Vis Perform. 2023;11.

2. Eckert KA, Carter MJ, Lansingh VC, Wilson DA. A Simple Method for Estimating the Economic Cost of Productivity Loss Due to Blindness and Moderate to Severe Visual Impairment. Ophthalmic Epidemiol. 2015;6586. doi:10.3109/09286586.2015.1066394

3. Smith TST, Frick KD, Holden BA, Naidoo KS. Potential lost productivity resulting from the global burden of uncorrected refractive error. Bull World Health Organ. 2009; 431–437. doi:10.2471/BLT.08.055673

4. Fred Hollows Foundation T. Bringing light to sight in Kenya The benefits of eliminating avoidable. 2014. Available: https://www.hollows.org/us/where-we-work/africa/kenya-2

5. Trading Economics. Kenya - Employment In Services (% Of Total Employment) - 2023 Data 2024 Forecast 1991-2020 Historical. 2023 p. 1. Available: https://tradingeconomics.com/kenya/employment-in-services-percent-of-total-employment-wb-data.html

6. Brunes A, Heir T. Visual impairment and employment in Norway. BMC Public Health. 2022;22: 648. doi:10.1186/s12889-022-13077-0

7. World Bank. Employment rate in Kenya. 2023 [cited 21 Jun 2023]. Available: https://www.ceicdata.com/en/indicator/kenya/employed-persons

8. Junankar PN. Labor Force Participation BT - Economics of the Labour Market: Unemployment and the Costs of Unemployment. In: Junankar PNR, editor. Economics of The Labour Market. London: Palgrave Macmillan UK; 2016. pp. 38–41. doi:10.1057/9781137555199_5

9. CEIC. Kenya Labour Force Participation Rate, 1990 – 2023 | CEIC Data. 2023 [cited 8 Dec 2023]. Available: https://www.ceicdata.com/en/indicator/kenya/labour-force-participation-rate

10. Whitfield R, Schwab L, Ross-degnan D, Steinkuller P, Swartwood J. Blindness and eye disease in Kenya : ocular status survey results from the Kenya Rural Blindness Prevention Pro ject. Br J Ophthalmol. 1990; 333–340.

11. Fricke TR, Holden BA, Wilson DA, Schlenther G, Naidoo KS, Resnikoff S, et al. Global cost of correcting vision impairment from uncorrected refractive error. Bull World Health Organ. 2012; 728–738. doi:10.2471/BLT.12.104034

12. International Agency for Prevention of. Ending avoidable sight loss. 2021. Available: https://www.iapb.org/wp-content/uploads/2022/02/2030inSight-Strategy-Document-Sep2021-English.pdf

13. Kenya Ministry of Health. National Eye Health Strategic Plan. 2020. Available: https://www.medbox.org/document/national-eye-health-strategic-plan-2020-2025

14. Kenya Ministry of Health. Scope of practice for ophthalmic workers. 2022. Available: https://www.health.go.ke/

15. Morjaria P, Ramson P, Gichangi M. Services for refractive error in Kenya. J Ophthalmol East Cent South Africa. 2013. Available: https://www.researchgate.net/publication/279926992

16. Morjaria P, Minto H, Ramson P, Gichangi M, Naidoo K, Gilbert C. Services for refractive error in Kenya : extent to which human resources and equipment are meeting VISION 2020 targets. J Ophthalmol East Cent South Africa. 2013;17: 44–49. Available: http://coecsa.org/ojs-2.4.2/index.php/JOECSA/article/view/78

17. International Agency for Prevention and Blindness. Core competency for the eye health workforce in the WHO African region. 2016. Available: https://www.iapb.org/wp-content/uploads/Core-Competencies-for-Eye-HWF-in-WHO-AFRO-Region.pdf

18. University of Nairobi. Department of Ophthalmology fee and funding. 2023 [cited 7 May 2023]. Available: https://opthalmology.uonbi.ac.ke/programs-content-type/master-medicine-ophthalmology

19. Patel I, West SK. Presbyopia : prevalence , impact , and interventions. Community Eye Heal J. 2007;20: 51–52.

20. KK Optic. What is the Cost of Prescriptive Eyeglasses in Kenya? - KK Opticians. 2022. Available: https://kkopticals.co.ke/

21. Lapaire Glasses. Quality & Affordable Eyeglasses in Africa | Lapaire Glasses. 2018 [cited 7 May 2023]. Available: https://lapaire.africa/
