# Supplementary material for: Estimation of the lost productivity to the GDP and the national cost of correcting visual impairment from refractive error in Kenya
Source: PLoS One. 2024 Mar 25;19(3):e0300799. doi: 10.1371/journal.pone.0300799 (PMC10962815; doi:10.1371/journal.pone.0300799)
Supplement: Attachment — Submitted filename: PONE-D-23-29248_reviewer final.pdf [file pone.0300799.s002.pdf]

PLOS ONE

Estimation of the lost productivity to the GDP and the national cost of correcting visual impairment from refractive error in Kenya

--Manuscript Draft--

**Manuscript Number:**	PONE-D-23-29248
**Article Type:**	Research Article
**Full Title:**	Estimation of the lost productivity to the GDP and the national cost of correcting visual impairment from refractive error in Kenya
**Short Title:**	Lost productivity and national cost of correcting visual impairment in Kenya

**Corresponding Author:**	Shadrack Muma<br>University of KwaZulu-Natal - Westville Campus<br>Kisumu, KENYA

**Keywords:**	productivity loss; gross domestic product; telemedicine; uncorrected refractive error, national costs; cost benefit analysis; productivity benefit

Abstract:

Background: In developing countries such as Kenya, minimal attention has been directed to population based studies on uncorrected refractive error (URE). However, in the absence of population based studies, other avenues are desirable to showcase to stakeholders in eye health the worth of addressing URE. Hence this study estimated the lost productivity to the Gross Domestic Product (GDP) as a result of URE and the national cost of correcting visual impairment from URE in Kenya.

Methods: The unadjusted GDP loss for the population aged 16-50 years and for population aged 16-60 years was calculated. Thereafter the unadjusted GDP loss for the population aged 16-50 years and 16-60 years as a product of the labour force participation rate (LFPR) and the employment rate (ER) was conservatively adjusted. The productivity loss of the caregivers of severe visual impaired persons was computed as a product of the average annual productivity for each caregiver and a 5% productivity loss due to visual impairment. Estimation of the national cost of addressing URE was based on spectacle provision cost, cost of training functional clinical refractionists and the cost of establishing refraction points. A cost benefit analysis was undertaken based on the national cost estimates and a factor of 3.5 times.

Results: The estimated unadjusted and the conservatively adjusted GDP loss among persons with URE in Kenya is approximately US$ 10,351,829 and US$ 2,945,249, respectively annually for population aged 16-60 years and US$ 9,404,043 and US$ 2,675,590 respectively for population aged 16-50 years. The productivity loss of caregivers for the severe visually impaired is approximately US$ 19,649,376 annually. Approximately US$ 214,274739 is required to provide corrective devices, US$ 95,092,500 to train clinical refractionists and US$ 39,800,000 to establish refraction points. The productivity benefit of correcting visual impairment is approximately US$ 2,976,138 annually.

Conclusion: The magnitude of productivity loss due to URE in Kenya is significant warranting prioritization of refractive error services by the government and all stakeholders as any investment directed towards addressing URE has the potential to yield a positive return.

**Order of Authors:**	Shadrack Muma
	Kovin Shunmugam Naidoo
	Rekha Hansraj

**Additional Information:**	
**Question**	**Response**
**Financial Disclosure**<br><br>Enter a financial disclosure statement that describes the sources of funding for the work included in this submission. Review	The author(s) received no specific funding for this work.

Powered by Editorial Manager® and ProduXion Manager® from Aries Systems Corporation

the submission guidelines for detailed requirements. View published research articles from *PLOS ONE* for specific examples.<br><br>This statement is required for submission and **will appear in the published article** if the submission is accepted. Please make sure it is accurate.<br><br>**Unfunded studies**<br>Enter: *The author(s) received no specific funding for this work.*<br><br>**Funded studies**<br>Enter a statement with the following details:<br>• Initials of the authors who received each award<br>• Grant numbers awarded to each author<br>• The full name of each funder<br>• URL of each funder website<br>• Did the sponsors or funders play any role in the study design, data collection and analysis, decision to publish, or preparation of the manuscript?<br>• **NO** - Include this sentence at the end of your statement: *The funders had no role in study design, data collection and analysis, decision to publish, or preparation of the manuscript.*<br>• **YES** - Specify the role(s) played.<br><br>* typeset	
**Competing Interests**<br><br>Use the instructions below to enter a competing interest statement for this submission. On behalf of all authors, disclose any competing interests that could be perceived to bias this work—acknowledging all financial support and any other relevant financial or non-financial competing interests.<br><br>This statement is required for submission and **will appear in the published article** if the submission is accepted. Please make sure it is accurate and that any funding sources listed in your Funding Information later in the submission form are also	The authors have declared that no competing interests exist.

declared in your Financial Disclosure statement.<br><br>View published research articles from *PLOS ONE* for specific examples.<br><br>**NO authors have competing interests**<br><br>Enter: *The authors have declared that no competing interests exist.*<br><br>**Authors with competing interests**<br><br>Enter competing interest details beginning with this statement:<br><br>*I have read the journal's policy and the authors of this manuscript have the following competing interests: [insert competing interests here]*<br><br>* typeset	
**Ethics Statement**<br><br>Enter an ethics statement for this submission. This statement is required if the study involved:<br><br>• Human participants<br>• Human specimens or tissue<br>• Vertebrate animals or cephalopods<br>• Vertebrate embryos or tissues<br>• Field research<br><br>Write "N/A" if the submission does not require an ethics statement.<br><br>General guidance is provided below. Consult the submission guidelines for detailed instructions. **Make sure that all information entered here is included in the Methods section of the manuscript.**	Maseno University ethics review Committee ref: MUERC/1051/22and Bimedical research ethics committee ref: BREC/ 00004105/2022<br>The ethics review Committee waived the requirement for informed consent.<br>This was a manuscript generated as part of the approved wider study.

Powered by Editorial Manager® and ProduXion Manager® from Aries Systems Corporation

**Format for specific study types**<br><br>**Human Subject Research (involving human participants and/or tissue)**<br>• Give the name of the institutional review board or ethics committee that approved the study<br>• Include the approval number and/or a statement indicating approval of this research<br>• Indicate the form of consent obtained (written/oral) or the reason that consent was not obtained (e.g. the data were analyzed anonymously)<br><br>**Animal Research (involving vertebrate animals, embryos or tissues)**<br>• Provide the name of the Institutional Animal Care and Use Committee (IACUC) or other relevant ethics board that reviewed the study protocol, and indicate whether they approved this research or granted a formal waiver of ethical approval<br>• Include an approval number if one was obtained<br>• If the study involved *non-human primates*, add *additional details* about animal welfare and steps taken to ameliorate suffering<br>• If anesthesia, euthanasia, or any kind of animal sacrifice is part of the study, include briefly which substances and/or methods were applied<br><br>**Field Research**<br><br>Include the following details if this study involves the collection of plant, animal, or other materials from a natural setting:<br>• Field permit number<br>• Name of the institution or relevant body that granted permission	
**Data Availability**<br><br>Authors are required to make all data underlying the findings described fully available, without restriction, and from the time of publication. PLOS allows rare exceptions to address legal and ethical concerns. See the PLOS Data Policy and FAQ for detailed information.	Yes - all data are fully available without restriction

Powered by Editorial Manager® and ProduXion Manager® from Aries Systems Corporation

A Data Availability Statement describing where the data can be found is required at submission. Your answers to this question constitute the Data Availability Statement and **will be published in the article**, if accepted.<br><br>**Important:** Stating 'data available on request from the author' is not sufficient. If your data are only available upon request, select 'No' for the first question and explain your exceptional situation in the text box.<br><br>Do the authors confirm that all data underlying the findings described in their manuscript are fully available without restriction?	
**Describe where the data may be found in full sentences. If you are copying our sample text, replace any instances of XXX with the appropriate details.**<br><br>• If the data are **held or will be held in a public repository**, include URLs, accession numbers or DOIs. If this information will only be available after acceptance, indicate this by ticking the box below. For example: *All XXX files are available from the XXX database (accession number(s) XXX, XXX.).*<br>• If the data are all contained **within the manuscript and/or Supporting Information files**, enter the following: *All relevant data are within the manuscript and its Supporting Information files.*<br>• If neither of these applies but you are able to provide **details of access elsewhere**, with or without limitations, please do so. For example:<br><br>*Data cannot be shared publicly because of [XXX]. Data are available from the XXX Institutional Data Access / Ethics Committee (contact via XXX) for researchers who meet the criteria for access to confidential data.*<br><br>*The data underlying the results presented in the study are available from (include the name of the third party*	The data will be presented upon reasonable request from the corrsponding author

and contact information or URL).<br>• This text is appropriate if the data are owned by a third party and authors do not have permission to share the data.<br><br>* typeset	
Additional data availability information:	

Powered by Editorial Manager® and ProduXion Manager® from Aries Systems Corporation

Manuscript
Click here to access/download;Manuscript;3. Cost estimates Manuscript Revised Plos One.docx

Estimation of the lost productivity to the GDP and the national cost of correcting visual impairment from refractive error in Kenya

Shadrack Muma[1¶], Kovin Shunmugam Naidoo[1, 2¶], Rekha Hansraj[1¶]

[1]Department of Optometry, College of Health Sciences, University of KwaZulu-Natal, Durban, South Africa

[2]OneSight EssilorLuxottica Foundation, South Africa

***** Corresponding author

Email- mumashadrack275@gmail.com (SM)

[¶]These authors contributed equally to this work.

Abstract

Background: In developing countries such as Kenya, minimal attention has been directed to population based studies on uncorrected refractive error (URE). However, in the absence of population based studies, other avenues are desirable to showcase to stakeholders in eye health the worth of addressing URE. Hence this study estimated the lost productivity to the Gross Domestic Product (GDP) as a result of URE and the national cost of correcting visual impairment from URE in Kenya.

Methods: The unadjusted GDP loss for the population aged 16-50 years and for population aged 16-60 years was calculated. Thereafter the unadjusted GDP loss for the population aged 16-50 years and 16-60 years as a product of the labour force participation rate (LFPR) and the employment rate (ER) was conservatively adjusted. The productivity loss of the caregivers of severe visual impaired persons was computed as a product of the average annual productivity for each caregiver and a 5% productivity loss due to visual impairment. Estimation of the national cost of addressing URE was based on spectacle provision cost, cost of training functional clinical refractionists and the cost of establishing refraction points. A cost benefit analysis was undertaken based on the national cost estimates and a factor of 3.5 times.

Results: The estimated unadjusted and the conservatively adjusted GDP loss among persons with URE in Kenya is approximately US$ 10,351,829 and US$ 2,945,249, respectively annually for population aged 16-60 years and US$ 9,404,043 and US$ 2,675,590 respectively for population aged 16-50 years. The productivity loss of caregivers for the severe visually impaired is approximately US$ 19,649,376 annually. Approximately US$ 214,274739 is required to provide corrective devices, US$ 95,092,500 to train clinical refractionists and US$ 39,800,000 to establish refraction points. The productivity benefit of correcting visual impairment is approximately US$ 2,976,138 annually.

Conclusion: The magnitude of productivity loss due to URE in Kenya is significant warranting prioritization of refractive error services by the government and all stakeholders as any investment directed towards addressing URE has the potential to yield a positive return.

Keywords: Productivity loss; gross domestic product; telemedicine; uncorrected refractive error, national costs; cost benefit analysis; productivity benefit

Introduction

The global burden of URE demands that all stakeholders in eye health direct efforts towards addressing URE for net economic gain [1–3]. Holistically, eye care professionals should be at the forefront in providing evidence on URE and showcase to the government in particular, why policy changes and actions should be undertaken to address URE. To achieve this in developing countries, eye care professionals in collaboration with other stakeholders should undertake population based studies and provide a national prevalence to policy makers for policy changes and recognition of the burden attributed to URE [1]. Even though evidence shows that URE impacts negatively on the quality of life [4], eye care professionals in Kenya have not shown the burden attributed to URE from population based studies, preventing the government of Kenya from allocating resources inclined towards addressing URE in Kenya. As a baseline, we intended to establish the lost productivity to the GDP as a result of URE and the national cost required to address visual impairment (VI) from URE in Kenya. It is anticipated that this estimate can act as a baseline and showcase to the government, eye care professionals and all stakeholders in eye health the need to direct more efforts towards addressing URE to achieve the 2030 IN SIGHT [5] which is intended to ensure accessibility, availability and affordability of refractive error (RE) services by 2030.

Materials and methods

The disability weights estimates of medium to severe visual impairment (MSVI) by Salomon et al [6] and the estimated prevalence of URE in Kenya [7] was adopted in calculating the unadjusted lost productivity to the Gross Domestic Product (GDP). Various assumptions were considered for the estimations. Firstly, the population below 16 years were excluded from the estimation with the assumption that they yield no economic productivity to a country GDP as

proposed by Smith et al. [9]. Secondly, the population with normal visual acuit[illegible]as included as they were assumed to have the potential of contributing to purchasing power parity adjusted GDP per capita of a country [9].

Using the consideration of 16-50 years as a productive age group [6, 8, 10], the unadjusted GDP loss was estimated as a product of the population above 16years and the disability weights of MSVI reported by Salomon et al. [6]. The disability weights estimates adopted for this study was 0.005 for mild visual impairment, 0.089 for moderate visual impairment and 0.314 for severe visual impairment [6]. Thereafter, the population above 50 years was excluded with an assumption that they contribute no productivity to the economy. Hence the computed unadjusted GDP loss for population aged 16-50 years was conservatively adjusted using the labour force participation rate (LFPR) of 0.739 and the employment rate (ER) of 0.385 in Kenya [11]. However, given that in Kenya the stipulated retirement age is 60 years [12], an assumption that the population aged 16-60 years contributes to a country GDP was made. Hence the unadjusted GDP loss for population aged 16-60 years was computed and conservatively adjusted using the labour force participation rate (LFPR) of 0.739 and the employment rate (ER) of 0.385 in Kenya [11].

The productivity benefit of addressing URE among visually impaired persons in Kenya of working age was estimated. The estimation adopted was that approximately 51% of the Kenyan population [illegible]e of working age [13] and the employment rate of visually impaired in Kenya is approximately 16% [14]. An assumption was made that if those with VI benefits from spectacles, then the employment rate will be equal to the national averag[illegible]15]. Hence the productivity benefit was estimated as a product of 51% of Kenyans of working age with VI and the employment rate of 38.5% for visually impaired in Kenya.

The productivity loss of the caregivers for severe visually impaired persons to the GDP was calculated based on various assumptions. The population aged 16-60 years was included with the assumption that they are productive and can take care of visually impaired persons. The average income of the ~~normal sighted~~ caregivers aged 16-60 years annually was calculated. Given that approximately 6.39% of Kenyans are visually impaired [7], an assumption was made that the population with severe VI would ideally require some sought of caregiving in the absence of corrective devices. Out of the 6.39% Kenyans with MSVI, it is estimated that 7.5% have severe VI [16]. Therefore, with a recommendation of a ratio of 1:1 ~~for a~~ caregiver to severe visually impaired persons, approximately 227,951 ~~caregivers are required~~. Based on a study by Fricke et al. [9], the assumption that each caregiver for a visually impaired person experiences a productivity loss of 5% to average productivity, was made. Hence the productivity loss of caregivers as a product of average annual productivity for each caregiver and the 5% productivity loss due to VI was computed.

For the national cost estimation, an estimation for the national cost required to establish ~~refraction~~ ~~points~~/vision centres, the national cost required to scale functional clinical refractionists and the national cost required to provide spectacles based on the estimated prevalence of URE of 6.39% in Kenya was computed [7]. With the assumption that URE cannot be addressed in the absence of adequate ~~refraction points~~ where ~~RE~~ patients can access RE services, the cost required to establish the refraction ~~points~~ in Kenya based on estimates from the IAPB of US$ 50,000 [5] and the Ministry of Health Kenya of US$ 100,000 [17] cost per ~~refraction point~~ was computed. The number of required ~~refraction points~~/vision centres was estimated based on some recommendations and assumptions. The first recommendation was that the establishment of one ~~refraction point~~ ~~to~~ serve a population of 50,000 with the assumption that

~~this will enhance quality delivery as~~ one functional clinical refractionist can attend ~~to~~ this population effectively. Although the recommendation of 1:50,000 would address issues around quality RE service delivery, ~~resources~~ constraints in developing countries such as Kenya was noted. As a result, a holistic conservative recommendation of one ~~refraction point~~ per 100,000 populations was made. The second recommendation ~~that~~ telemedicine integration should be prioritized given limited human resource available in Kenya was made.

On the national cost estimate required to scale human resource calculations were conducted based on different ratios to project on the number of functional clinical refractionists that will be required. The calculation was to provide a more holistic ratio to be adopted based on the available resources within ~~the~~ Kenya~~n context.~~ Based on the various roles that each cadre is destined to play in the eye care industry in Kenya as per the scope of practice for ophthalmic workers, an assumption that ophthalmologists may spend roughly 20% of their time doing refraction, ophthalmic clinical officers roughly 30% of their time while ophthalmic nurses may spend ~~roughly~~ 15% of their time was made [18]. This assumption was intended to provide a justifiable ground for stakeholders in eye health to direct efforts on scaling clinical refractionists in eye health. The number of required functional clinical refractionists from the existing eye care ~~cadres~~ having the potential to conduct refraction in Kenya based on assumptions from a study by Morjaria et al. [20] was estimated. The deficit of functional clinical refractionists based on the World Health Organization (WHO) conservative recommendation of 1:50,000 was computed. With assumptions that not all eye care professionals having the potential to undertake refraction ~~do so,~~ a recommendation of 1:10,000 to address this aspect under strengthened telemedicine integration was made.

Finally, based on the national cost required to provide the 6.39% individuals with URE in Kenya

with spectacles [7], a review of the current private sector rates, as well as those of social enterprises (SE) was undertaken with an aim of estimating the cost required to provide RE services for the population in dire need of these in Kenya. The public health sector in Kenya was excluded as they do not offer spectacles instead referring patients to the private sector for spectacles while SE located within the public sector were included such as the Kenya Society for the Blind. To estimate the number of individuals with URE who already have corrective devices, the 6% estimates of the number of spectacles wearers in Tanzania was adopted [21]. The estimated number of individuals with URE but already have corrective devices were excluded from the national cost estimates. Using the estimated prevalence of 6.39% of URE in Kenya, the national cost for correcting URE in Kenya using average spectacles charges from the private sector and the SE sectors was computed.

After the national cost estimates for addressing URE in Kenya, a cost benefit analysis was conducted to show whether it would be worth investing the total amount required to provide corrective devices, scaling human resource and establishing refraction points/vision centres in Kenya. Considering that the benefit of addressing URE remains significant and mostly overrides cost invested in addressing URE by a factor of 3.5 times [13], the implication is that if one dollar is invested in addressing URE then a possible return of US$ 3.56 will be accrued. Hence the cost benefit analysis was computed as a product of the cumulative national cost required to address URE and the factor of 3.5 times. The estimate was intended to showcase whether it is worth investing the national cost of addressing URE by stakeholders in eye health in Kenya.

This study was approved by Maseno University Ethics Review Committee, Kenya ref: MUERC/1051/22 and the Biomedical Research Ethics Committee, South Africa ref: BREC/00004105/2022. The ethics review committee waived the requirements for the informed

consent

Results

Lost productivity of visually impaired persons to the GDP

With around 27,826,973 males and females aged 16 years and above in Kenya [22], the estimated unadjusted GDP loss in Kenya was computed as a product of 27,826,973 for the population 16 years and above with the MSVI estimates of 0.005 for mild impairment, 0.089 for moderate impairment and 0.314 for severe impairment by Salomon et al., [6]. The unadjusted GDP loss was estimated at approximately US$ 139,134 for mild impairment, US$ 2,476,600 for moderate visual impairment and US$ 8,737,669 for severe visual impairment. The cumulative unadjusted GDP loss for the population 16 years and above as a result of MSVI in Kenya is approximately US$ 11,353,403.

Population aged 16-50 years

The conservative estimate for the population aged 16-50 years in Kenya is approximately 23,049,130 as per the 2019 census [23]. The unadjusted and the adjusted GDP loss from MSVI in Kenya were computed as shown in Table 1.

Table 1: Unadjusted and the adjusted GDP loss due to visual impairment for the population aged 16-50 years in Kenya

Magnitude of visual impairment	Population aged 16-50 years	Disability weights	Unadjusted GDP loss (US$)	Labour force participation rate (LFPR)	Employment rate (ER)	Adjusted GDP loss (US$)
Mild	23,049,130	0.005	115,245	0.739	0.385	32,788
Moderate	23,049,130	0.089	2,051,372	0.739	0.385	583,646

Severe	23,049,130	0.314	7,237,426	0.739	0.385	2,059,156
**Total**			9,404,043			2,675,590

Population aged 16-60 years

With the assumption that the population aged 60 ~~years~~ ~~are~~ productive to the country's GDP, the conservative estimate for the population aged 16-60 years in Kenya which is approximately 25,372,134 as per the 2019 census [23]. The unadjusted and adjusted GDP loss from MSVI in Kenya was computed as shown in Table 2.

Table 2: Unadjusted and adjusted GDP loss due to visual impairment for the population aged 16-60 years in Kenya

**Magnitude of visual impairment**	**Population aged 16-60 years**	**Disability weights**	**Unadjusted GDP loss (US$)**	**Labour force participation rate (LFPR)**	**Employment rate (ER)**	**Adjusted GDP loss (US$)**
Mild	25,372,134	0.005	126,860	0.739	0.385	36,093
Moderate	25,372,134	0.089	2,258,119	0.739	0.385	642,468
Severe	25,372,134	0.314	7,966,850	0.739	0.385	2,266,688
**Total**			10,351,829			2,945,249

Lost productivity by caregivers of visually impaired persons to the GDP

The average income for a caregiver of the visually impaired person in Kenya is approximately US$ 1,724 annually [24]. Hence for the estimated 227,951 caregivers for the severe VI individuals, the income will be approximately US$ 392,987,524 annually. Therefore, for a single

caregiver using the 5% productivity loss as a result of taking care of the severe visually impaired individuals, approximately US$ 86.2 is lost annually. Hence cumulatively a total of US$ 19,649,376 is lost annually if the 7.5% of severe visually impaired persons receive care from the normal sighted population.

National cost estimates for addressing uncorrected refractive error in Kenya

Based on proportions of cadres who undertake refraction by Morjaria et al. [20] in Kenya and our assumption on the number of hours each cadre may allocate for refraction [18], we estimated that approximately 560 functional clinical refractionists exist in Kenya. Based on various ratios and the recommendation of 1:10,000, currently there is a deficit of 4,196 functional clinical refractionists in Kenya as shown in Table 3.

Table 3: Deficit of functional clinical refractionists required in Kenya based on different practitioner – population ratios

Recommendations	Required functional clinical refractionists	Existing functional clinical refractionists	Deficit
1:250,000	190	560	Exceeds
1:100,000	475	560	Exceeds
1:50,000	951	560	391
1:25,000	1,942	560	1,382
1:10,000	4,756	560	4,196

The current spectacle charges from the private sector in Kenya is estimated at an average cost of US$ 75 [25] and that of social enterprises (SE) an average of US$ 35 [26]. Hence, with the current rates, majority of the Kenyan population are unable to afford the spectacles as only

38.7% are employed [14]. In consideration of the estimated prevalence of URE in Kenya of 6.39% equivalent to 3,039,358 [7], the estimated national cost for addressing URE using the private sector rate of US$ 75 was approximately US$ 227,951,850 while the estimated national cost based on SE rates of US$ 35 is approximately US$ 106,377,530. With the assumption that there is a population already having corrective devices, we adopted the estimates from Tanzania in which 6% of the population with URE are estimated to have spectacles already [21]. Excluding the population already having corrective devices, we estimated the national cost for addressing URE based on the private sector rate as approximately US$ 214,274,739 while the estimated national cost based on SE rates was approximately US$ 99,994,878.

National cost of training human resource to address uncorrected refractive error in Kenya

Currently, the cost of training an eye care professional in Kenya is approximately US$ 1,680 - US$ 20,450 [27]. Hence if human resource is to be scaled based on various ratios, then approximately US$ 1,103,760- US$ 95,092,500 will be required to train the human resources for eye health as shown in Table 4.

Table 4: Estimated cost of training human resource in eye health in Kenya based on cadres and recommended ratios

Cadre	Cost (US$) of training to completion	Source	Existing functional clinical refractionists	Total cost (US$) to train for 1:50,000 recommendation	Total cost (US$) to train for 1:25,000 recommendation	Total cost (US$) to train for 1:10,000 recommendation
Optometrist	7,000	[28]	178	5,411,000	12,348,000	32,046,000
Ophthalmologist	20,450	[27]	106	17,280,250	37,546,200	95,092,500
Ophthalmic Clinical Officer/CS	3,004	[29]	164	2,364,148	5,341,112	13,794,368

Optometry tec[illegible]ologist	1,680	[29]	294	1,103,760	2,768,640	7,496,160
Ophthalmic nurse	3,004	[29]	80	2,616,484	5,593,448	14,046,704

Conventionally, if one vision centre/~~refraction point~~ can be established to serve a population of 50,000, then approximately 951 functional ~~refraction points~~ will be required to serve the Kenyan population conveniently given that currently there are approximately 49,456,894 individuals in Kenya. Using the IAPB recommendation of an average of US$50,000 for equipping and establishing a vision centre [30] and the Kenya Ministry of Health, [31] estimate of US$100,000, the estimated cost required to establish the ~~refraction points~~ in Kenya are shown in Table 5.

Table 5: Estimated cost of establishing the required refraction points/vision centres in Kenya

**~~Refraction points/~~V[illegible] per population**	**Required ~~refraction points~~**	**Existing ~~refraction points~~**	**Deficit ~~refraction points~~**	**Cost of establishing the refraction points**	
				**IAPB Recommended rates (US$)**	**Ministry of Health, Kenya rates (US$)**
1:100,000	475	77	398	19,900,000	39,800,000
1:50,000	951	77	874	43,700,000	87,400,000

Productivity benefit to correcting the visually impaired persons in Kenya

The productivity benefit was computed as 51% of the 6.39% of Kenyans with URE (T[illegible]ng Economics, 2023; Muma, 2023). The proportion of the working age was found to be

approximately 1,[illegible],072. Using the employment rate of 38.5% [14], the productivity benefit for addressing URE in Kenya will be approximately US$ 2,976,138 annually based on the current dollar exchange rates.

Cost benefit analysis

The cost benefit analysis estimate was computed as a product of national cost estimates on the area of investments and the factor of 3.5 times as reported by Fred Hollows Foundation for Kenya [13]. The result is shown in Table 6.

Table 6: Cost benefit analysis for investments towards addressing uncorrected refractive error in Kenya

**Area of investment**	**National cost estimate required (US$)**	**Factor estimate**	**Return on investment (US$)**
Human resource	95,092,500	3.5	332,823,750
Corrective devices	214,274,739	3.5	749,961,586
~~Refraction points/~~vision centres	39,800,000	3.5	139,300,000

Discussion

The growth of an economy's GDP could be determined by the population within a specific age group having the potential to undertake activities inclined towards net economic gain. However, this can only be achieved if the population~~s~~ with disabilities are provided with the right devices to address their conditions limiting them from undertaking their daily activities. Through the conservative approach, ~~our~~ study estimates ~~report~~ a lost productivity of approximately US$ 919,042 annually to the GDP as a result of URE. Similarly, for caregivers unadjusted lost productivity of approximately US$ 246,273,100 to the GDP is recorded annually. This justifies

that assertion that URE contributes to the country GDP loss warranting the government to direct more efforts towards addressing URE. With the limited resources [32–34] during the COVID-19 pandemic, the government allocated resources to address the pandemic, a clear indication that evidence on the severity of a condition may force the government to take action and address the situation. Hence the burden of URE in Kenya should be showcased to the government by eye care professionals and other stakeholders to justify why attention should be directed towards addressing URE. However, it appears that most eye care professionals in developing countries such as Kenya remain unaware that it is their role to undertake population based studies and showcase the burden attributed to URE to the government and stakeholders in eye health for an action. As it stands, the government and other stakeholders in eye health are directing efforts towards addressing URE based on global assumptions and estimates since a clear picture of the burden of URE in developing countries such as Kenya remains unknown. Therefore, the estimates provided in this paper are intended to act as a baseline to showcase to stakeholders in eye health the need for action to address URE.

~~Even though~~ URE contributes negatively to the GDP [35], dispensing a simple pair of spectacles not only impacts on the quality of life of an individual, but contributes to net economic gain [36]. Our study has shown that a productivity benefit of correcting VI in Kenya is approximately US$ 2,976.138 annually. This justifies the value of providing spectacles and other corrective devices to patients with RE. However, with approximately 59.6% to 85.7% of Kenyans living on less than US$ 1.9 a day [37], it will be necessary for the government to integrate spectacles and other corrective devices within the public sector so as to assist patients with URE to gain access and afford the available RE services. More holistically, designing an approach where corrective devices are provided at prices based on the average income of everyone across the economic

pyramid is desirable. This is to ensure that patients with RE become productive and to the GDP since any investment on URE contributes a return with a factor of 3.5 times [13].

Uncorrected RE remains the leading cause of VI globally [38] and Kenya is not an exception. As a result, eye care professionals should be at the forefront in advocating for the inclusion of eye health within the public sector. Although the constitution of Kenya 2010 stipulates that the government should ensure access to quality healthcare [39], they are incapacitated with the private sector dominating the eye health sector. However, with the current charges for spectacles in the private sector [25], the majority of the population across the economic pyramid cannot afford the spectacles primarily as the poverty rate ranges between 59.6%-85.7% with the majority living on less than US$ 1.9 a day [37]. Therefore, achieving an effective RE coverage within the Kenyan context will require partly that the government recognize the efforts that various stakeholders are making towards addressing URE. Tactically, considering the relatively reasonable charges by the SE, the government should establish policies to allow the SE, which are organizations that participate in business ventures through a commercial approach in order to fulfill a social purpose [40], to introduce RE services within the existing public sector institutions so as to scale effective RE coverage in a more cost effective and sustainable way.

While the human resource remains a major challenge within the eye care ecosystem in developing countries such as Kenya [41], a needs based approach of training human resource integrated with telemedicine which is the mode of healthcare delivery through information and technology, is desirable [42, 43]. This study has shown that that there are approximately 822 eye care providers who undertake refraction in Kenya. However, not all of them meet the threshold of functional clinical refractionists who are destined to undertake refraction one hundred percent of their time. As a result, out of the 822 eye care providers who undertake refraction,

approximately 560 meet the threshold of functional clinical various roles each cadre is destined to undertake [18]. Therefore, if the WHO conservative recommendation of 1:50,000 is adopted as suggested by Fricke et al., [9] for Kenya, then an additional 391 functional clinical refractionists are required. However, this study argues that for effective RE coverage, a conventional recommendation of 1:10,000 is desirable and if adopted, 4,196 clinical refractionists will be trained at a cost of approximately US$ 95,092,500. Therefore, considering the limited resources and the high cost of training for eye care professionals who end up not operating as functional clinical refractionists, needs based training should focus on optometrists and integrating the telemedicine to scale effective RE coverage in Kenya.

In Kenya, there are approximately 12,393 health facilities, with only 77 having functional optical units [20]. This low number of ~~refraction points~~ in Kenya is linked to the lack of a national prevalence from population based studies which can showcase to the government the burden of URE and the need for action in this regard. However, for effective RE coverage to be achieved, ~~refraction points/~~vision centres are desirable. This study estimates that approximately 951 vision centres/~~refraction points~~ are required for effective RE service delivery. This implies that one ~~refraction point~~ is desirable per 50,000 ~~populations~~. With limited resources in Kenya, a holistic approach of one r~~efraction point~~ per 100,000 is desirable warranting 475 ~~refraction points~~ in Kenya at a cost of US$ 39,800,000 based on the Ministry of Health estimate per ~~refraction point~~ of US$100,000 [17]. The establishment of the ~~refraction points~~ is costly; however, a cost benefit analysis still shows that a return will be accrued if such ~~refraction points~~ are established. Therefore, a technological approach like telemedicine should be integrated within the established optical units to scale RE service delivery.

Addressing URE should conventionally be prioritized ~~for~~ quality of life. Hence considering the

current situation in which approximately 83% of VI is present in low-income and middle-income countries among the underserved population [44, 45], the productivity loss might be huge given the limited estimates showing the cost benefit analysis of URE. As a result, this paper will act as baseline for stakeholders in eye health especially the government to understand the value of investing the estimated national cost for addressing URE in Kenya. Addressing URE will not only be beneficial to the individuals but will also benefit the caregivers of individuals with URE and contribute to the economic growth of a country. Our study has denoted that any investment directed towards addressing URE contribute to a positive return. As a result, it is worthy of attention for stakeholders in eye health sector to invest more in addressing URE as a projected positive return will be accrued provided the investment is properly executed by professionals.

In conclusion, URE contributes to lost productivity and reduction of the GDP in Kenya, therefore investing the national cost ~~for scaling~~ human resource, ~~refraction points~~ and providing spectacles to the population in dire need will contribute a productivity benefit. Although the estimates in this study may not be exact due to aspects like varying exchange rates for a dollar, to the best of our knowledge, this is the first study to be conducted in Kenya on the lost productivity to the GDP as a result of URE. It also provides an overview of the current situation in Kenya ~~with respect to URE~~ highlighting why investments towards addressing URE should be prioritized by the government and stakeholders in eye health.

Acknowledgement

The authors are grateful ~~for~~ the statistician who contributed in analysis.

References

[1] Hashemi H, Fotouhi A, Yekta A, et al. Global and regional estimates of prevalence of refractive errors: Systematic review and meta-analysis. *J Curr Ophthalmol* 2018; 30: 3–

22.

[2] Burton MJ, Ramke J, Marques AP, et al. The Lancet Global Health Commission on Global Eye Health: vision beyond 2020. *The Lancet Global Health* 2021; 9: e489–e551.

[3] Narayan S. *Addressing the Unmet Need: An Analysis of the Global Prevalence of Refractive Error and its Possible Solutions*. Portland, ~~OR~~. Epub ahead of print 1 January 2014. DOI: 10.15760/honors.87.

[4] Kandel H, Khadka J, Shrestha MK, et al. Uncorrected and corrected refractive error experiences of Nepalese adults: a qualitative study. *Ophthalmic Epidemiol* 2018; 25: 147–161.

[5] IAPB. *ENDING AVOIDABLE SIGHT LOSS*, https://www.iapb.org/wp-content/uploads/2022/02/2030inSight-Strategy-Document-Sep2021-English.pdf (2021).

[6] Salomon JA, Haagsma JA, Davis A, et al. Disability weights for the Global Burden of Disease 2013 study. *Lancet Glob Heal* 2013; 712–723.

[7] Muma S. Estimation of the Prevalence of Refractive Error in Kenya: A Systematic Review and Meta-Analysis. *Optom Vis Perform*.

[8] Smith TST, Frick KD, Holden BA, et al. Potential lost productivity resulting from the global burden of uncorrected refractive erro[illegible]009; 431–437.

[9] Fricke TR, Holden BA, Wilson DA, et al. Global cost of correcting vision impairment from uncorrected refractive erro[illegible]012; 728–738.

[10] Naidoo KS, Fricke TR, Frick KD, et al. Potential Lost Productivity Resulting from the Global Burden of Myopia: Systematic Review, Meta-analysis, and Modeling. *Ophthalmology* 2019; 126: 338–346.

[11] World Bank. Employment rate in Kenya,

https://www.ceicdata.com/en/indicator/kenya/employed-persons (2023, accessed 21 June 2023).

[12] LOF Kenya. *THE CONSTI[illegible]TION OF KENYA, 2010*. 2010.

[13] Fred Hollows Foundation T. *Bringing light to sight in Kenya The benefits of eliminating avoidable*. 2014.

[14] Trading Economics. Kenya - Employment In Services (% Of Total Employment) - 2023 Data 2024 Forecast 1991-2020 Historical. 2023; 1.

[15] Taylor HR, Pezzullo ML, Nesbitt SJ, et al. Costs of Interventions for Visual Impairment. *Am J Ophthalmol* 2007; 143: 561-565.e1.

[16] Whitfield R, Schwab L, Ross-degnan D, et al. Blindness and eye disease in Kenya : ocular status survey results from the Kenya Rural Blindness Prevention Project.

[17] Kenya Ministry of Health. *National Eye Health Strategic Plan*. 2020

[18] MoH. *Scope of practice for ophthalmic workers*. 2022.

[19] Morjaria P, Ramson P, Gichangi M. *Services for refractive error in Kenya: extent to which human resources and equipment are meeting VISION 2020 targets myopia control View project Nigeria national blindness and low vision survey project View project East African Journal of Ophthalmology S*, https://www.researchgate.net/publication/279926992 (2013).

[20] Morjaria P, Ramson P, Gichangi M. *Services for refractive error in Kenya: extent to which human resources and equipment are meeting VISION 2020 targets myopia control View project Nigeria national blindness and low vision survey project View project East African Journal of Ophthalmology Services for refractive error in Kenya: extent to which human resources and equipment are meeting VISION 2020 targets*,

https://www.researchgate.net/publication/279926992 (2013).

[21] Patel I, West SK. Presbyopia : prevalence , impact , and interventions. *Community Eye Heal J* 2007; 20: 51–52.

[22] Census. *2019 KENYA POPULATION AND HOUSING CENSUS VOLUME I : POPULATION BY COUNTY AND SUB-COUNTY*. 2019.

[23] KNBS. *2019 Kenya Population and Housing Census*. 2019.

[24] World Salaries. Average Caregiver Salary in Kenya for 2023, https://worldsalaries.com/average-caregiver-salary-in-kenya/ (2023, accessed 12 August 2023).

[25] OPTIC KK. What is the Cost of Prescriptive Eyeglasses in Kenya? - KK Opticians, https://kkopticals.co.ke/what-is-the-cost-of-prescriptive-eyeglasses-in-kenya/ (2022).

[26] Lapaire. Quality & Affordable Eyeglasses in Africa | Lapaire Glasses, https://en-kn.lapaire.africa/?shpxid=078246a2-ebd1-41a2-8a97-116bd360b101 (2018).

[27] UoN. MASTER OF MEDICINE IN OPHTHALMOLOGY | Department of Ophthalmology, https://opthalmology.uonbi.ac.ke/programs-content-type/master-medicine-ophthalmology (2023).

[28] MMUST. *MASINDE MULIRO UNIVERSITY OF SCIENCE AND TECHNOLOGY OFFICE OF THE REGISTRAR , ACADEMIC AFFAIRS FEES STRUCTURE FOR 2018 / 2019 ACADEMIC YEAR SCHOOL OF PUBLIC HEALTH & BIOMEDICAL TECHNOLOGY (SPHBT) National Bank of Kenya , Kaka mega Branch KCB Bank , Kaka*. 2019.

[29] ~~KMTC.~~ Kenya Medical Training College | Training For Better Health, https://kmtc.ac.ke/ (2022).

[30] IAPB. Strategy for the Elimination of Vision Impairment from Uncorrected Refractive Error - The International Agency for the Prevention of Blindness, https://www.iapb.org/learn/resources/strategy-for-the-elimination-of-vision-impairment-from-uncorrected-refractive-error/ (2022).

[31] Kenya Ministry of Health. *National Strategic Plan For Eye Health and Blindness Preventio[illegible]12 to 2018*. 2011.

[32] Courtright P, Mathenge W, Kello AB, et al. Setting targets for human resources for eye health in sub-Saharan Africa: What evidence should be used? *Human Resources for Health*; 14. Epub ahead of print 2016. DOI: 10.1186/s12960-016-0107-x.

[33] Palmer JJ, Chinanayi F, Gilbert A, et al. Mapping human resources for eye health in 21 countries of sub-Saharan Africa: Current progress towards VISION 2020. *Hum Resour Health*; 12. Epub ahead of print 15 August 2014. DOI: 10.1186/1478-4491-12-44.

[34] Yorston D, Buchan J. Investment in human resources improves eye health for all. *Community Eye Heal J* 2018; 35: 37–39.

[35] Khan AA, Khan NU, Bile KM, et al. *Creating synergies for health systems strengthening through partnerships in Pakistan – a case study of the national eye health programme*. 2008.

[36] Marques AP, Ramke J, Cairns J, et al. The economics of vision impairment and its leading causes: A systematic review. *eClinicalMedicine* 2022; 46: 101354.

[37] World Bank. *Macro Poverty Outlook for Kenya*. 2022.

[38] W[illegible]O. World report on vision. *World Heal Organ* 2019; 214: 1–160.

[39] Government of Kenya (GoK). *Kenya ’ s Constitution of 2010*. 2010.

[40] Luke B, Chu V. Social enterprise versus social entrepreneurship : An examination of the ‘

why ’ and ‘ how ’ in pursuing social change. *Int Small Bus J* 2013; 31: 765–766.

[41] Palmer J, Chinanayi F, Gilbert A, et al. Mapping human resources for eye health in 21 countries of sub-Saharan Africa: current progress towards VISION 2020. *Hum Resour Health*; 12. Epub ahead of print 15 August 2014. DOI: 10.1186/1478-4491-12-44.

[42] Haleem A, Javaid M, Singh RP, et al. Telemedicine for Healthcare : Capabilities , features , barriers , and applications ~~Telemedicine for healthcare : Capabilities , features , barriers , and applications.~~ *Sensors Int* 2021; 2: 100117.

[43] Kim T, Zuckerman JE. Realizing the potential of telemedicine in global health. 9. Epub ahead of print 2019. DOI: 10.7189/jogh.09.020307.

[44] Ulldemolins AR, Lansingh VC, Valencia LG, et al. Review Article Social inequalities in blindness and visual impairment : A review of social determinants. Epub ahead of print 2011. DOI: 10.4103/0301-4738.100529.

[45] Zhang JH, Ramke J, Jan C, et al. Review Advancing the Sustainable Development Goals through improving eye health : a scoping review. *Lancet Planet Heal* 2022; 270–280.
