# Supplementary material for: Estimation of the lost productivity to the GDP and the national cost of correcting visual impairment from refractive error in Kenya
Source: PLoS One. 2024 Mar 25;19(3):e0300799. doi: 10.1371/journal.pone.0300799 (PMC10962815; doi:10.1371/journal.pone.0300799)
Supplement: Attachment — Submitted filename: PONE-D-23-29248_R1 Final.pdf [file pone.0300799.s004.pdf]

PLOS ONE

Estimation of the lost productivity to the GDP and the national cost of correcting visual impairment from refractive error in Kenya

--Manuscript Draft--

**Manuscript Number:**	PONE-D-23-29248R1
**Article Type:**	Research Article
**Full Title:**	Estimation of the lost productivity to the GDP and the national cost of correcting visual impairment from refractive error in Kenya
**Short Title:**	Lost productivity and national cost of correcting visual impairment in Kenya
**Corresponding Author:**	Shadrack Muma<br>University of KwaZulu-Natal - Westville Campus<br>Kisumu, KENYA
**Keywords:**	productivity loss; gross domestic product; telemedicine; uncorrected refractive error, national costs; Cost benefit analysis; productivity benefit
**Abstract:**	Background: In developing countries such as Kenya, minimal attention has been directed towards population based studies on uncorrected refractive error (URE). However, [illegible]e absence of population based studies, other avenues are desirable to showcase [illegible] stakeholders in eye health the worth of addressing URE. Hence this study estimated the lost productivity to the Gross Domestic Product (GDP) as a result of URE and the national cost required to address visual impairment from URE in Kenya.<br>Methods: The lost productivity to the GDP for the population aged 16-60 years was calculated. Thereafter the productivity loss of the caregivers of severe visual impaired individuals was computed as a product of the average annual productivity for each caregiver and a 5% productivity loss due to visual impairment. The productivity benefit of correcting refractive error was estimated based on the minimum wage for individuals aged 16-60 years with URE. Estimation of the national cost of addressing URE was based on spectacle provision cost, cost of training functional clinical refractionists and the cost of establishing vision centres. A cost benefit analysis was undertaken based on the national cost estimates and a factor of 3.5 times.<br>Results: The estimated lost productivity to the GDP due to URE in in Kenya is approximately US$ 671,455,575 -US$ 1,044,486,450 annually for population aged 16-60 years. The productivity loss of caregivers for the severe visually impaired is approximately US$ 13,882,899 annually. Approximately US$ 246,750,000 is required to provide corrective devices, US$ 413,280- US$ 108,262,300 to train clinical refractionists and US$ 39,800,000 to establish vision centres. The productivity benefit of correcting visual impairment is approximately US$ 41,126,400 annually. Finally, a cost benefit analysis showed a return of US$ 378,918,050 for human resource, US$ 863,625,000 for corrective devices and US$ 139,300,000 for establishment of vision centres.<br>Conclusion: The magnitude of productivity loss due to URE in Kenya is significant warranting prioritization of refractive error services by the government and all stakeholders since any investment directed towards addressing URE has the potential to yield a positive return.
**Order of Authors:**	Shadrack Muma
	Kovin Shunmugam Naidoo
	Rekha Hansraj
**Response to Reviewers:**	Dear Dr. Ogbomo (Academic Editor – Plos One)<br>Detailed below are the revisions made to the article titled ‘Estimation of the lost productivity to the GDP and the national cost of correcting visual impairment from refractive error in Kenya (Manuscript. No. PONE-D-23-29248)’. The changes were made based on the comments/suggestions of Reviewer #1, Reviewer #2, Reviewer #3 and the Academic Editor. All changes have been tracked on the revised manuscript. The authors’ responses to queries received are formatted through word track changes.<br>ACADEMIC EDITOR:<br>Before acceptance please ensure the following has been addressed:

Powered by Editorial Manager® and ProduXion Manager® from Aries Systems Corporation

	This is noted<br>Address the queries of each review as clearly as possible.<br>This is noted and has been addressed across the document<br>Undertake extensive English Language edit of the entire manuscript<br>Editing has been undertaken across the document<br>Reviewer #1: Thank you for your submission. The paper is relevant and highlights the importance of accessibility to vision services and optical corrective devices and the inequality that exists across the world. However, review of the paper with a recommendation of major review, is required.<br>Please see the main points below and detail comments in the manuscript:<br>As English is not your first language the paper would benefit form a proof-reading by a native speaker or a proof-reading service. Some sentences are formulated in an unclear fashion or are incomplete.<br>This is noted and has been addressed across the document as suggested in the reviewed manuscript<br>Try to avoid terms like cadres or refraction points. Alternatives have been provided in comments in the manuscript.<br>This is noted. Changes have been made across the document as suggested<br>References should be reviewed and formatted in accordance to the publishers guidance. Some of them are incomplete, some have invalid links attached.<br>Organisations such as WHO should be written in full in reference list.<br>This has been addressed as suggested<br>In body of the manuscript, when referring to organisations, e.g. WHO and others, first mention name in full, then abbreviate.<br>Thanks. This has been revised accordingly across the document<br>Some of the estimations and calculations could be explained clearer.<br>This is noted and has been addressed across the document. Line 80-264<br>The various ratios and recommendations should be referenced.<br>Yes the recommendations have been referenced. Line 153-156, 159-162, 237-238, 262-263<br>Think about how you would integrate telemedicine in your vision centres as in optometry, it has a limited role and can be used for triaging the patient, obtaining basic history 7 symptoms and for follow ups with the patient. This also would require a infrastructure (phones, internet for video calls with patient and other colleagues). If you are referring to telemedicine hubs and for eyecare professionals to communicate between themselves in regards to a patient/patient presentation or course of treatment or management, please specify that. 144-146<br>This is noted. “If you are referring to telemedicine hubs and for eyecare professionals to communicate between themselves in regards to a patient/patient presentation or course of treatment or management”. Yes this is what we referred to. Detailed on page 148-150<br>Reviewer #2: I find the topic interesting and important to the eye care services in Kenya. From my point of view, the paper needs to be revised and improved.<br>This is noted and has been addressed as suggested<br>First, the authors don´t provide a definition for Refractive error. That is a critical point since that will have an impact on the age group that should be considered to estimate productivity losses.<br>The definition has been provided in line 54-55<br>It is unclear why they decided to use two age groups 16-50 and 16-60.<br>This is noted. We would like to clarify that the initial estimates was based on the first study on global estimate of the cost required to address URE and the lost productivity to the GDP. However, with the advancement in the approaches used, we have made the calculations using the latest development (A Simple Method for Estimating the Economic Cost of Productivity Loss Due to Blindness and Moderate to Severe Visual Impairment by Eckert and colleagues). Therefore, this study adopts the 16-60 years only. Details are provided in line 80-92.<br>Why should people above the age of 50 y.o be considered unproductive if the retirement age in the country is 60 y.o.<br>The unproductive age in the study has been rectified as individuals below 16 years and those above 60 years. Line 91<br>They also don´t provide a definition for labour force participation and employment rate which I believe would be useful to do.<br>This is noted. The definitions have been provided in line 108-113<br>Second, they use disability weights to measure productivity losses without providing a

Powered by Editorial Manager® and ProduXion Manager® from Aries Systems Corporation

	good rational to do it. Disability weights and productivity losses should not be seen as substitutes. They measure different aspects of life. Disability weights represent the magnitude of health loss while productivity losses represent "the production loss due to illness, disability and death of productive persons, both paid and unpaid".<br>This is noted. Based on the current estimate approach, the disability weights were not used instead the minimum wage, the affected population and reduced earnings was used as proposed in a study by Eckert and colleagues: A Simple Method for Estimating the Economic Cost of Productivity Loss Due to Blindness and Moderate to Severe Visual Impairment. Line 80-92 provides the details<br>Third, it is not clear why are they using the all population (either 16-50 or 16-60) and not just the population who have refractive error (which will vary with the prevalence rate of refractive error in Kenya).<br>This is noted. The current estimate is based on the 3.5 million Kenyans with URE. Line 86-92 details<br>Forth, I think there is a mistake with the reference of the disability weights used in the paper. Salomon´s et al (ref 6) report disability weights of 0.003 for mild visual impairment, 0.031 for moderate visual impairment and 0.184 for severe visual impairment. The disability weights used in the paper are proposed by WHO. Authors should explain why they did not use the latest disability weights figures.<br>This is noted. The disability weight approach has been eliminated with adoption of the latest formula for estimating the productivity loss. Therefore there is shift from the initial formula to the latest. Details are provided in the methodology section line 80-211<br>Fifth, there is something wrong with table 1 and table 2 (and with the way productivity losses are estimated). The product between number of people (Column 1 population aged 16-50 years) and disability weights (Column 2 population aged 16-50 years) can not directly produce a value expressed in USD.<br>We agree and based on the current formula, we have made changes in line 213-226<br>Finally, the national cost estimation and cost benefit analysis are not well describe and needs to be revised.<br>This has been revised as proposed in line 287-305 and line 227-272 in the revised document<br>The reference list doesn´t follow common reference styles.<br>This is noted and has been addressed as instructed<br>Reviewer #3: The study is interesting and makes significant contribution to the economics of uncorrected refractive errors in Kenya<br>The study will require language editing<br>This is noted and we have edited across the document<br>Clarify how the average income of the normal sighted caregiver aged 16 – 60 years was calculated<br>The minimum wage in Kenya is approximately US$ 101.505. Given that approximately 7.5% of the individuals with URE in Kenya have severe VI, we computed the annual income with an assumption that they constitute the LFPR. Details are provided on line 219-226<br>Line 130 – 135. Why was the assumption of time spent in doing refraction leave out the cadre of optometrists?<br>Optometrists are core and in this study we consider them as functional clinical refractionists. The WHO outline that optometrists are well placed to address URE. Line 230-232 provides the estimates.<br>Line 135 – 139. Are optometrists classified under the clinical refractionist cadre? Please explain to provide clarity. Yes, the details are in line 166-167<br>Referencing style should follow journal's guidelines.<br>This is noted and has been addressed under references sub-heading
**Additional Information:**	
**Question**	**Response**
**Financial Disclosure**<br><br>Enter a financial disclosure statement that describes the sources of funding for the work included in this submission. Review the submission guidelines for detailed requirements. View published research	The author(s) received no specific funding for this work.

articles from *PLOS ONE* for specific examples.<br><br>This statement is required for submission and **will appear in the published article** if the submission is accepted. Please make sure it is accurate.<br><br>**Unfunded studies**<br>Enter: *The author(s) received no specific funding for this work.*<br><br>**Funded studies**<br>Enter a statement with the following details:<br>• Initials of the authors who received each award<br>• Grant numbers awarded to each author<br>• The full name of each funder<br>• URL of each funder website<br>• Did the sponsors or funders play any role in the study design, data collection and analysis, decision to publish, or preparation of the manuscript?<br>• **NO** - Include this sentence at the end of your statement: *The funders had no role in study design, data collection and analysis, decision to publish, or preparation of the manuscript.*<br>• **YES** - Specify the role(s) played.<br><br>* typeset	
**Competing Interests**<br><br>Use the instructions below to enter a competing interest statement for this submission. On behalf of all authors, disclose any competing interests that could be perceived to bias this work—acknowledging all financial support and any other relevant financial or non-financial competing interests.<br><br>This statement is required for submission and **will appear in the published article** if the submission is accepted. Please make sure it is accurate and that any funding sources listed in your Funding Information later in the submission form are also declared in your Financial Disclosure statement.	The authors have declared that no competing interests exist.

View published research articles from *PLOS ONE* for specific examples.<br><br>**NO authors have competing interests**<br><br>Enter: *The authors have declared that no competing interests exist.*<br><br>**Authors with competing interests**<br><br>Enter competing interest details beginning with this statement:<br><br>*I have read the journal's policy and the authors of this manuscript have the following competing interests: [insert competing interests here]*<br><br>* typeset	
**Ethics Statement**<br><br>Enter an ethics statement for this submission. This statement is required if the study involved:<br><br>• Human participants<br>• Human specimens or tissue<br>• Vertebrate animals or cephalopods<br>• Vertebrate embryos or tissues<br>• Field research<br><br>Write "N/A" if the submission does not require an ethics statement.<br><br>General guidance is provided below. Consult the submission guidelines for detailed instructions. **Make sure that all information entered here is included in the Methods section of the manuscript.**	Maseno University ethics review Committee ref: MUERC/1051/22and Bimedical research ethics committee ref: BREC/ 00004105/2022<br>The ethics review Committee waived the requirement for informed consent.<br>This was a manuscript generated as part of the approved wider study.

Powered by Editorial Manager® and ProduXion Manager® from Aries Systems Corporation

**Format for specific study types**<br><br>**Human Subject Research (involving human participants and/or tissue)**<br>• Give the name of the institutional review board or ethics committee that approved the study<br>• Include the approval number and/or a statement indicating approval of this research<br>• Indicate the form of consent obtained (written/oral) or the reason that consent was not obtained (e.g. the data were analyzed anonymously)<br><br>**Animal Research (involving vertebrate animals, embryos or tissues)**<br>• Provide the name of the Institutional Animal Care and Use Committee (IACUC) or other relevant ethics board that reviewed the study protocol, and indicate whether they approved this research or granted a formal waiver of ethical approval<br>• Include an approval number if one was obtained<br>• If the study involved *non-human primates*, add *additional details* about animal welfare and steps taken to ameliorate suffering<br>• If anesthesia, euthanasia, or any kind of animal sacrifice is part of the study, include briefly which substances and/or methods were applied<br><br>**Field Research**<br><br>Include the following details if this study involves the collection of plant, animal, or other materials from a natural setting:<br>• Field permit number<br>• Name of the institution or relevant body that granted permission	
**Data Availability**<br><br>Authors are required to make all data underlying the findings described fully available, without restriction, and from the time of publication. PLOS allows rare exceptions to address legal and ethical concerns. See the PLOS Data Policy and FAQ for detailed information.	Yes - all data are fully available without restriction

A Data Availability Statement describing where the data can be found is required at submission. Your answers to this question constitute the Data Availability Statement and **will be published in the article**, if accepted.<br><br>**Important:** Stating 'data available on request from the author' is not sufficient. If your data are only available upon request, select 'No' for the first question and explain your exceptional situation in the text box.<br><br>Do the authors confirm that all data underlying the findings described in their manuscript are fully available without restriction?	
**Describe where the data may be found in full sentences. If you are copying our sample text, replace any instances of XXX with the appropriate details.**<br><br>• If the data are **held or will be held in a public repository**, include URLs, accession numbers or DOIs. If this information will only be available after acceptance, indicate this by ticking the box below. For example: *All XXX files are available from the XXX database (accession number(s) XXX, XXX.*).<br>• If the data are all contained **within the manuscript and/or Supporting Information files**, enter the following: *All relevant data are within the manuscript and its Supporting Information files.*<br>• If neither of these applies but you are able to provide **details of access elsewhere**, with or without limitations, please do so. For example:<br><br>*Data cannot be shared publicly because of [XXX]. Data are available from the XXX Institutional Data Access / Ethics Committee (contact via XXX) for researchers who meet the criteria for access to confidential data.*<br><br>*The data underlying the results presented in the study are available from (include the name of the third party*	All relevant data are within the manuscript and its Supporting Information files.

and contact information or URL).<br>• This text is appropriate if the data are owned by a third party and authors do not have permission to share the data.<br><br>* typeset	
Additional data availability information:	

Manuscript
Click here to access/download;Manuscript;Manuscript.docx

Estimation of the lost productivity to the GDP and the national cost of correcting visual impairment from refractive error in Kenya

Shadrack Muma[1¶], Kovin Shunmugam Naidoo[1, 2¶], Rekha Hansraj[1¶]

[1]Department of Optometry, College of Health Sciences, University of KwaZulu-Natal, Durban, South Africa

[2]OneSight EssilorLuxottica Foundation, Paris, France

***** Corresponding author

Email- mumashadrack275@gmail.com (SM)

¶These authors contributed equally to this work.

Abstract

Background: In developing countries such as Kenya, minimal attention has been directed towards population based studies on uncorrected refractive error (URE). However, in the absence of population based studies, other avenues are desirable to showcase to stakeholders in eye health the worth of addressing URE. Hence this study estimated the lost productivity to the Gross Domestic Product (GDP) as a result of URE and the national cost required to address visual impairment from URE in Kenya.

Methods: The lost productivity to the GDP for the population aged 16-60 years was calculated. Thereafter the productivity loss of the caregivers of severe visual impaired individuals was computed as a product of the average annual productivity for each caregiver and a 5% productivity loss due to visual impairment. The productivity benefit of correcting refractive error was estimated based on the minimum wage for individuals aged 16-60 years with URE. Estimation of the national cost of addressing URE was based on spectacle provision cost, cost of training functional clinical refractionists and the cost of establishing vision centres. A cost benefit analysis was undertaken based on the national cost estimates and a factor of 3.5 times.

Results: The estimated lost productivity to the GDP due to URE in in Kenya is approximately US$ 671,455,575 -US$ 1,044,486,450 annually for population aged 16-60 years. The productivity loss of caregivers for the severe visually impaired is approximately US$ 13,882,899 annually. Approximately US$ 246,750,000 is required to provide corrective devices, US$ 413,280- US$ 108,262,300 to train clinical refractionists and US$ 39,800,000 to establish vision centres. The productivity benefit of correcting visual impairment is approximately US$ 41,126,400 annually. Finally, a cost benefit analysis showed a return of US$ 378,918,050 for human resource, US$ 863,625,000 for corrective devices and US$ 139,300,000 for establishment of vision centres.

Conclusion: The magnitude of productivity loss due to URE in Kenya is significant warranting prioritization of refractive error services by the government and all stakeholders since any investment directed towards addressing URE has the potential to yield a positive return.

Keywords: Productivity loss; gross domestic product; telemedicine; uncorrected refractive error, national costs; cost benefit analysis; productivity benefit

Introduction

Refractive error which is a type of vision problem that makes it hard to see clearly is the leading cause of visual impairment (VI) globally [1]. According to the Global Burden of Disease 2020, 338.8 million people globally had moderate and severe visual impairment (MSVI) with a projection of 535 million by 2050 if no interventions are taken to reduce avoidable blindness [2]. As a result, all stakeholders in eye health should direct efforts towards addressing URE for net economic gain [3–5]. Holistically, eye care professionals should be at the forefront in providing evidence on URE and showcase to the government in particular, why policy changes and actions should be undertaken to address URE. In Kenya it is estimated that approximately 3.5 million individuals suffers from RE warranting the need for actions to address this condition [6]. To achieve this in developing countries such as Kenya, eye care professionals in collaboration with other stakeholders such as social entrepreneurs, Ministry of Health and commercial entrepreneurs should support population based studies. The population based studies would showcase the actual burden and influence the policy makers to establish policies and recognize the importance of addressing the burden attributed to URE [3]. ~~Even though~~ evidence shows that URE impacts negatively on the quality of life [7] standard operating procedures and central monitoring should be in place to showcase to the government the burden attributed to URE from population based studies so as to influence the allocation of resources to address this condition in Kenya. The financial impact of vision loss on the global economy has been a major concern over the past decade [8–13]. As a result, showcasing the financial impact attributed to URE is desirable to influence the policy makers on the need for an action. Therefore, this study intended to establish the lost productivity to the GDP as a result of URE and the national cost required to address visual impairment (VI) from URE in Kenya. It is anticipated that this estimate can act as a baseline and showcase to the government, eye care professionals and all stakeholders in eye

health the worth of directing efforts towards addressing accessibility, availability and affordability of refractive error (RE) services to achieve the 2030 IN SIGHT [14]

Materials and methods

According to Eckert and colleagues [15], MSVI results in a range of reduced productivity with 30%-55% not working while 45%-70% have their earning reduced by 35%. In Kenya, the minimum wage (MW) which is the lowest remuneration that employers can legally pay their employee is approximately US$ 101.505 per month [16]. The annual MW for the 45%-70% of the individuals with URE who are working was computed. Thereafter, a factor of 0.35 was used to estimate the reduced earnings for the visually impaired individuals who are working. Currently in Kenya, approximately 3.5 million people are estimated to suffer from RE [6]. The method proposed by Eckert and colleagues [15] in calculating the lost productivity as a result of MSVI was adopted. The productivity loss was computed as a product of (0.45 of the affected population * 0.35 of reduced earnings) and (0.7 of the affected population * 0.35 of reduced earnings) [15]. The population aged 16-60 years with VI was included for the estimations with the assumption that the population below 16 years and those above 60 years with URE yield no economic productivity to a country's GDP as proposed by Smith and colleagues [17].

The productivity benefit of addressing URE among visually impaired individuals in Kenya of working age (16-60 years) was estimated. In Kenya, approximately 51% of the visually impaired individuals constitute the working age group [18] with an ER of 16% [19]. In the absence of VI, those treated would be employed at the same rate as the individuals with good vision [20]. In Kenya, the employment rate is estimated at 38.5% nationally for the population aged 16-60 years with a MW of US$ 101.505 monthly [19]. To scale the current ER for visually impaired individuals to the national average of 38.5%, additional investments beyond the recurrent

expenditure in eye health care to develop sustainable primary and secondary healthcare systems are desirable. An assumption was made that if individuals with VI benefits from spectacles and low vision devices, then the ER would potentially be scaled to the national average of 38.5% [19]. An assumption was also made that if the visually impaired individuals who have attained the working age are provided with corrective devices and employed thereafter then the productivity benefit would increase. The productivity benefit was computed in three perspectives. Firstly, the productivity benefit for the 51% of the individuals who are visually impaired and have attained the employment age was computed as a product of the annual MW and the population [21]. The unadjusted productivity benefit was adjusted using the LFPR which is the estimate of the economy's active workforce [22]. In Kenya, the LFPR is approximately 0.739 [23]. Secondly, the current productivity benefit accrued from the 16% of the visually impaired in Kenya was calculated. Finally, a productivity benefit was computed with an assumption that the ER which is the measure of the extent to which available labour resources are being utilized for visually impaired is scaled to the national average of 38.5% [22].

The productivity loss of the caregivers for severe visually impaired individuals to the GDP was computed. The population aged 16-60 years with normal vision was included with the assumption that they are productive and can take care of visually impaired individuals. The average income of the normal sighted caregivers aged 16-60 years annually was calculated. Given that approximately 3.5 million Kenyans are visually impaire[illegible], an assumption was made that the population with severe VI would require some sought of care in the absence of corrective devices. Out of the 3.5 million Kenyans with MSVI, it is estimated that approximately 7.5% have severe VI [24]. Therefore, it was assumed that there is one caregiver for every individual with severe VI. The number of required caregivers was estimated based on the number of

individuals with severe VI in Kenya. Based on a study by Fricke and colleagues [25] , it was assumed that every caregiver for a visually impaired individual experiences a productivity loss of 5% on the total average yearly productivity. Hence the productivity loss of caregivers was computed as a product of the average annual productivity for each caregiver and the 5% productivity loss due to the care provided. It was assumed that if severe VI is addressed, it is expected that caregivers will be able to increase their productivity through employment, education and other approaches.

The national cost calculation entailed summation of the cost required to establish vision centres (VCs), the cost required to scale functional clinical refractionists and the cost required to provide spectacles based on the estimated prevalence of URE in Kenya. An assumption was made that URE cannot be addressed effectively in the absence of adequate VCs where patients with RE can access RE services. The cost required to establish the VCs in Kenya was computed based on estimates from the IAPB of US$ 50,000 [14] and the Ministry of Health of Kenya ~~of~~ US$ 100,000 [26] per vision centre ~~(VC)~~. The number of required VCs was estimated based on the population density and the Ministry of Health in Kenya consideration in establishing a healthcare facility [26]. In Kenya, the Ministry of Health recommends establishment of health facilities in an area with a population density of 100,000 [26]. However, the services offered in such healthcare facilities may not be inclusive of RE services. Therefore, this paper recommended integration of VCs within the existing healthcare facilities to offer RE services to a population of 100,000. This paper also recommended that in the presence of resources, one VC should serve a population of 50,000 with an assumption that this will enhance quality RE services delivery. Given the limited resources in Kenya to scale the VCs [27], This paper proposed a holistic conservative recommendation of one VC per 100,000 populations based on the Ministry of

Health criteria for establishing healthcare facility [26]. The study also recommended that telemedicine integration into the eye health ecosystem should be adopted to ensure that the limited human resource available in Kenya could scale service delivery to the VCs. The proposed telemedicine should entail a communication among eye care professionals in regards to a patient presentation and course of management.

The national cost estimate required to scale human resource was undertaken. The calculations conducted were based on the Ministry of Health recommendations on the ratios of human resource to population in Kenya [26]. The recommendations by the Ministry of Health on eye care professionals to population includes; ophthalmologis[illegible]:250,000, optometrists 1:250,000, ophthalmic clinical officers 1:100,000, optometry technologists 1:100,000, ophthalmic clinical officers/cataract surgeons 1:125,000 and ophthalmic nurses 1: 100,000 [28]. Even though the scope of practice for ophthalmic workers in Kenya allows the above categories of eye care professionals to undertake refraction, not all meets the threshold of a functional clinical refractionist who undertakes refraction 100% of their time [29]. The World Health Organization (WHO) recommendations on the minimum eye care teams for one per million population includes ophthalmologists=4, optometrists=10, primary healthcare workers=200 and allied health professionals=10 [30]. The key activities proposed by the WHO recognize the potential of optometrists in meeting the threshold for a functional clinical refractionist [30]. Based on the range of services that ophthalmic workers in Kenya undertakes as per their scope of practice [29], this paper estimated that ophthalmologists may spend roughly 20% of their time doing refraction, ophthalmic clinical officers roughly 30% of their time, optometrists may spend approximately 90% of their time and finally ophthalmic nurses may spend roughly 15% of their time [29]. This estimation was intended to provide a justifiable ground for stakeholders in eye

health to direct efforts towards scaling eye care professionals meeting the threshold for functional clinical refractionists in Kenya. The number of required functional clinical refractionists from the existing eye care professionals having the potential to conduct refraction in Kenya was calculated based on the estimates by Morjaria and collegues [32]. The deficit of functional clinical refractionists was computed based on the WHO recommendation of one functional clinical refractionist per 100,000 populations [30]. Even though the WHO recommendation is ideal, this paper presents different proportions that could be achieved through integration of technological approaches such as telemedicin[illegible] As a result, this study estimated the number of functional clinical refractionists that should be trained to undertake refraction if adequate resources are available and telemedicine integration is prioritized. The calculations were based on the ratio of one refractionist per 50,000, 25,000 and 10,000 population. This study recommended that if one refractionist per 50,000, 25,000 and 10,000 population ratios are to be adopted then strengthened telemedicine integration is desirable. The estimated cost for training the deficit of functional clinical refractionists was computed based on the existing each eye care professional in Kenya who operates as functional clinical refractionists. The cost required to train functional clinical refractionists was computed for each category of the eye care professional based on the deficit and the recommendations by the WHO, Ministry of Health and this study recommendations based on telemedicine integration.

Finally, based on the national cost required to provide the 3.5 million individuals with URE in Kenya with spectacles[6], a review of the current private sector rates, as well as those of social enterprises (SE) was undertaken with an aim of estimating the cost required to provide RE services for the population in dire need of these in Kenya. The public health sector in Kenya was excluded as they do not offer spectacles but instead referring patients to the private sector for

spectacles. The SE located within the public sector such as the Kenya Society for the Blind were included. To estimate the number of individuals with URE who already have corrective devices, the 6% (210,000) estimates of the number of spectacles wearers in Tanzania was adopted [33]. The estimated number of individual with URE already having corrective devices was excluded from the national cost estimates. The national cost for correcting URE for the 3.5 million Kenyans was computed based on the average spectacles charges from the private sector and the SE sectors.

After the national cost estimates for addressing URE in Kenya were provided, a cost benefit analysis was conducted to show whether it would be worth investing the total amount required to provide corrective devices, scaling human resources and establishing VCs in Kenya. Considering that the benefit of addressing URE remains significant and mostly overrides cost invested in addressing URE by a factor of 3.5 times [18], the implication is that if one dollar is invested in addressing URE ~~then a possible~~ return of US$ 3.56 ~~will be accrued.~~ Hence the cost benefit analysis was computed as a product of the cumulative national cost required to address URE and the factor of 3.5 times was used. The estimate was intended to showcase whether it is worth investing the national cost of addressing URE by stakeholders in eye health in Kenya.

This study was approved by Maseno University Ethics Review Committee in Kenya reference MUERC/1051/22 and the Biomedical Research Ethics Committee in South Africa reference BREC/00004105/2022. The ethics review committee waived the requirements for the informed consent. This waiver was attributed to the nature of data recorded which is available within the public domains with no human subject involved [34].

Results

Lost productivity of visually impaired individuals to the GDP

The annual MW for the 45%-70% of the working individuals with MSVI in Kenya is approximately US$ 1,918,444,500 –US$ 2,984,247,000. The estimated productivity loss to the GDP as a result of VI in Kenya is approximately US$ 671,455,575 -US$ 1,044,486,450 annually.

Lost productivity by caregivers of visually impaired individuals to the GDP

The number of caregivers required to attend to the 7.5% Kenyans with severe VI is approximately 227,951 caregivers. The average income for a caregiver of the visually impaired person in Kenya is approximately US$ 101.505 monthly [16]. Hence the annual MW for the estimated 227,951 caregivers is approximately US$ 277,657,995. Therefore, if the 5% productivity loss as a result of taking care of the severe visually impaired individuals is adopted, then approximately US$ 60.90 per caregiver is lost annually. Hence cumulatively a total of US$ 13,882,899 is lost annually if the 7.5% of severe visually impaired individual receive care from the healthy population.

National cost estimates for addressing uncorrected refractive error in Kenya

Based on the scope of practice for ophthalmic workers allowed to undertake refraction in Kenya [29], the estimates on the proportions of professionals who undertake refraction by Morjaria and colleagues [31] in Kenya and this study assumption is that ophthalmologists, ophthalmic clinical officers, optometrists and ophthalmic nurses may spend approximately 20%, 30%, 90% and 15% of their time respectively doing refraction [29]. This study estimated that approximately 560 functional clinical refractionists exist in Kenya. Based on this study recommendation of one functional clinical refractionist per 10,000 populations if telemedicine is integrated, 4,756 functional clinical refractionists will be required in Kenya as shown in Table 1.

Table 1: Deficit of functional clinical refractionists required in Kenya based on different

practitioner – population ratios

Recommendations	Source	Required functional clinical refractionists	Existing functional clinical refractionists	Deficit
1:250,000	[35]	190	560	Exceeds
1:100,000	[29]	475	560	Exceeds
1:50,000	[36]	951	560	391
1:25,000	[37]	1,942	560	1,382
1:10,000	[28]	4,756	560	4,196

The current spectacle charges from the private sector in Kenya is estimated at an average cost of US$ 75 [38] and that of SE an average of US$ 35 [39]. Hence, with the current rates, majority of the Kenyan population are unable to afford the spectacles as only 38.5% are employed [19]. In consideration of the estimated prevalence of URE in Kenya of 3.5 million[6], the estimated national cost for addressing URE using the private sector rate of US$ 75 is approximately US$ 262,500,00 while the estimated national cost based on SE rates of US$ 35 is approximately US$ 122,500,00. With the assumption that there is a population already having corrective devices, this study adopted the estimates from Tanzania in which 6% (210,000) of the population with URE are estimated to have spectacles already[40]. The population with RE who already have corrective devices was excluded from the estimates. Out of the 3.5 million individuals with RE in Kenya, approximately 210, 000 already have corrective devices. Therefore, the estimated national cost for addressing URE based on the private sector rate was approximately US$ 246,750,000 while the estimated national cost based on SE rates was approximately US$ 115,150,000.

National cost of training human resource to address uncorrected refractive error in Kenya

Currently, the cost of training an eye care professional in Kenya is approximately US$ 1,680 -

US$ 20,450 [41]. Hence if human resource is to be scaled based on various ratios recommended by the WHO, the Ministry of Health and this study recommendations, then approximately 540 functional clinical refractionists will be required for 1:100,000, 1,080 functional clinical refractionists for 1:50,000, 2,160 functional clinical refractionists for 1:25,000 and 5,400 functional clinical refractionists for 1:10,00[illegible] The cost required to train the professionals based on the ratios above will be approximately US$ 413,280- US$ 108,262,300. Details are shown in Table 2.

Table 2: Estimated cost of training human resource in eye health in Kenya based on professionals and proposed ratios

				**Total cost (US$) for training functional clinical refractionist; n=required number**			
**Professionals**	**Cost (US$) of training to completion**	**Source**	**Existing functional clinical refractionists**	**Required (n=540) 1:100,000** [29]	**Required (n=1,080) 1:50,000** [36]	**Required (n=2,160) 1:25,000** [37]	**Required (n=5,400) 1:10,000** [28]
Optometrist	7,000	[42]	178	2,534,000	6,314,000	13,874,000	36,554,000
Ophthalmologist	20,450	[41]	106	8,875,300	19,918,300	42,004,300	108,262,300
Ophthalmic Clinical Officer/CS	3,004	[43]	164	1,129,504	2,751,664	5,995,984	15,728,944
Optometry technologist	1,680	[43]	294	413,280	1,320,480	3,134,880	8,578,080
Ophthalmic nurse	3,004	[43]	80	1,381	3,004,000	6,248,320	15,981,280

Conventionally, if one VC is established to serve a population of 50,000, then approximately 1,080 functional VCs will be required to serve the Kenyan population conveniently given that currently the Kenyan population is approximately 54 million [44]. Using the International Agency for Prevention and Blindness (IAPB) recommendation of an average of US$50,000 for equipping and establishing a VC [45] and the Kenya Ministry of Health, [46] estimate of

US$100,000, the estimated cost required to establish the VCs in Kenya are shown in Table 3.

Table 3: Estimated cost of establishing the required VCs in Kenya

VCs per population	Required VCs	Existing VCs	Deficit VCs	Cost of establishing the VCs	
				IAPB Recommended rates (US$)	Ministry of Health, Kenya rates (US$)
1:100,000	475	77	398	19,900,000	39,800,000
1:50,000	951	77	874	43,700,000	87,400,000

Productivity benefit to correcting the visually impaired individuals in Kenya

To scale the ER for visually impaired to the national average of 38.5%, additional 22.5% of the visually impaired should be employed. Out of the 3.5 million individuals in Kenya with VI [6], 1,785,000 have attained the employment age. Considering the MW of US$ 101.505 monthly [21], the unadjusted productivity benefit will be approximately US$ 2,174,237,100 annually if the 51% of the visually impaired individuals ~~within~~ 16-60 years are employed. This estimate was considered exceptional given that not all visually impaired individuals may meet the thresholds and requirements to be employed. As a result, the unadjusted productivity benefit was adjusted using the LFPR of 0.739. The adjusted productivity benefit is approximately US$ 1,606,761,216 annually. The second productivity benefit currently generated by the 16% of the visually impaired in Kenya is approximately US$ 41,126,400 annually. Finally, it was assumed that if the ER for visually impaired individuals is scaled to the national average of 38.5% then the productivity benefit will be approximately US$ 98,960,400 annually based on the current dollar exchange rates.

Cost benefit analysis

The estimation of the cost benefit analysis was based on three aspects. The first aspect was the

human resource. Based on this aspect, a cost benefit analysis was undertaken to show whether the cost of US$ 108,262,300 required in scaling the human resource would yield a positive return worth the investment. The number of the required human resources was determined based on the Ministry of Health recommendation of 1:100,000 and this study recommendation of 1:50,000, 1:25,000 and 1:10,000 for professional to the population. The second aspect was provision of corrective devices. This study computed a cost benefit analysis to show whether investing the cost of US$ 246,750,000 in providing corrective devices to the 3.5 million individuals with RE in Kenya was wort[illegible]The final aspect was the VCs establishment. This study determined the cost benefit analysis to show whether it is worth investing US$ 39,800,000 to establish VCs in Kenya. For each US$1 invested in the efforts to eliminate VI in Kenya, a return of US$3.56 could potentially be realized [18]. The cost benefit analysis estimate was computed as a product of the national cost estimates for scaling human resource, establishing VCs and providing corrective devices and the factor of 3.5 times. The result is shown in Table 4.

Table 4: Cost benefit analysis for investments towards addressing uncorrected refractive error in Kenya

Area of investment	National cost estimate required (US$)	Factor estimate	Return on investment (US$)
Human resources	108,262,300	3.5	378,918,050
Corrective devices	246,750,000	3.5	863,625,000
Vision centres	39,800,000	3.5	139,300,000

Discussion

The growth of an economy's GDP could be determined by the population aged 16-60 years having the potential to undertake activities inclined towards enhancing net economic gain [47]. However, this can only be achieved if the population with disability is provided with the right devices to address their condition which limits them from undertaking their daily activities. Through the conservative approach, this study estimates a lost productivity of approximately US$ 671,455,575 -US$ 1,044,486,450 annually to the GDP as a result of URE. Similarly, for caregivers, the lost productivity to the GDP is estimated at approximately US$ 13,882,899 annually. This justifies the assertion that URE contributes to the country GDP loss warranting the government to direct more efforts towards addressing URE. With the limited resources [48–50] during the COVID-19 pandemic, the government allocated resources to address the pandemic, a clear indication that evidence on the severity of a condition may force the government to take action and address the situation. Hence the burden of URE in Kenya should be showcased to the government by eye care professionals and other stakeholders to justify why attention should be directed towards addressing URE. However, there is lack of standard operating procedures and central monitoring as a minimum warranting the need for collaboration between the eye care professionals, researchers and all stakeholders in eye health. As it stands, the government and other stakeholders in eye health are directing efforts towards addressing URE based on global assumptions and estimates since a clear picture of the burden of URE in developing countries such as Kenya remains unknown. Therefore, the estimates provided in this paper are intended to act as a baseline to showcase to stakeholders in eye health the need for action to address URE.

Uncorrected refractive error contributes negatively to the GDP [51]. ~~However,~~ [illegible]pensing a simple pair of spectacles not only impacts on the quality of life of an individual, but contributes

to net economic gain [52]. This study shows that the productivity benefit of correcting VI in Kenya is approximately US$ 41,126,400 annually. This justifies the value of providing spectacles and other corrective devices to patients with RE. However, with approximately 59.6% to 85.7% of Kenyans living on less than US$ 1.9 a day [53], it would be necessary for the government to integrate spectacles and other corrective devices within the public sector ~~so as~~ to assist patients with URE to gain access and be able to afford the available RE services. More holistically, designing an approach where corrective devices are provided at prices based on the average income of everyone across the economic pyramid is desirable. This is to ensure that patients with RE become productive and contribute to the GDP since any investment on URE contributes a return of a factor of 3.5 times [18].

Uncorrected RE remains the leading cause of VI globally [54] and Kenya is not an exception [6]. As a result, eye care professionals in collaboration wit[illegible]akeholders in eye health should be at the forefront in advocating for the inclusion of eye health within the public sector. Although the constitution of Kenya 2010 stipulates that the government should ensure access to quality healthcare [55], it is incapacitated with the private sector dominating the eye health sector. With the current charges for spectacles in the private secto[illegible]8], the majority of the population across the economic pyramid cannot afford the spectacles ~~primarily,~~ as the poverty rate ranges between 59.6%-85.7% with the majority living on less than US$ 1.9 a day [53]. Therefore, achieving an effective RE coverage will require ~~partly~~ the governmen[illegible] recognize the efforts that various stakeholders are making towards addressing URE. Tactically, considering the relatively reasonable charges by the SE, the government should establish policies to allow the SE, which are organizations that participate in business ventures through a commercial approach in order to fulfill a social purpose [56], to introduce RE services within the existing public sector institutions

so as to scale effective RE coverage in a more cost effective and sustainable way.

Human resource development is a fundamental concept in a health system and capacity building strategies should be built on the community requirements [57]. While the human resources remains a major challenge within the eye care ecosystem in developing countries such as Kenya [36], a needs based approach of training professionals together with integrated telemedicine which is the mode of healthcare delivery through information and technology, is desirable [58,59]. Telemedicine has been applied within remote settings to overcome geographical barriers to healthcare access, providing an alternative means of connecting patients to specialists [60]. A meeting abstract by Randhawa and colleagues [61] examining telehealth delivered subjective refraction found no statistically significant difference between in person and telehealth delivered subjective refraction. This study has shown that that there are approximately 822 eye care providers who undertake refraction in Kenya. However, not all of them meet the threshold of functional clinical refractionists who undertake refraction one hundred percent of their time. As a result, out of the 822 eye care providers who undertake refraction, approximately 560 meet the threshold of functional clinical refractionists while others engage on various roles such as screening for eye diseases, eye surgery, training and treatment [29]. Therefore, if the recommendation of 1:50,000 is adopted as suggested by Fricke and colleagues [25] in Kenya, then an additional 391 functional clinical refractionists are required. However, this study estimates that for effective RE coverage, a conventional recommendation of 1:10,000 is desirable and if adopted, 4840 clinical refractionists should be trained at a cost of approximately US$ 108,262,300. Therefore, considering the limited resources and the high cost of training for eye care professionals who end up not operating as functional clinical refractionists, needs based training should be used and focus on optometrists and integrating the telemedicine to scale effective RE

coverage in Kenya.

In Kenya, there are approximately 12,393 health facilities, with only 77 having functional optical units [31]. This low number of units in Kenya is linked to the lack of a demonstrated national prevalence of URE from population based studies which can showcase to the government the burden of URE and the need for action in this regard. ~~However,~~ for effective RE coverage to be achieved, the establishment of VCs are desirable. This study estimates that approximately 951 VCs are required for effective RE service delivery. This implies that one VC is desirable per 50,000. With limited resources in Kenya, a holistic approach of one VC per 100,000 is desirable warranting 475 of such units to be established in Kenya at a cost of US$ 39,800,000 based on the Ministry of Health of Kenya estimate of funding per VC of US$100,000 [26]. The establishment of the VCs is costly; however, a cost benefit analysis still shows that a return will be accrued if such units are established. Therefore, a technological approach like telemedicine should be integrated within the established optical units to scale RE service delivery.

Addressing URE should conventionally be prioritized for quality life. Hence considering the current situation in which approximately 83% of VI is present in low-income and middle-income countries among the underserved population [62,63], the productivity loss might be huge given the limited estimates showing the cost benefit analysis of URE. As a result, this paper will act as baseline for stakeholders in eye health especially the government to understand the value of investing the estimated national cost for addressing URE in Kenya. Addressing URE will not only be beneficial to the individuals but will also benefit the caregivers of individuals with URE and contribute to the economic growth of a country. This study has showed that any investment directed towards addressing URE contribute to a positive return. As a result, it is worthy of attention for stakeholders in eye health sector to invest more in addressing URE as a projected

positive return will be accrued provided the investment is properly executed by professionals.

In conclusion, URE contributes to lost productivity and reduction of the GDP in Kenya, therefore investing the national cost in training human resources, establishing VCs and providing spectacles to the population in dire need will contribute a productivity benefit. Although the estimates in this study may not be exact due to aspects like varying exchange rates for a dollar, to the best of our knowledge, this is the first study to be conducted in Kenya on the lost productivity to the GDP as a result of URE. It also provides an overview of the current situation in Kenya highlighting why investments towards addressing URE should be prioritized by the government and stakeholders in eye health.

Acknowledgement

The authors would like to acknowledge the statistician for assisting during data analysis.

References

1. Dandona R, Dandona L. Refractive error blindness. Bull World Health Organ. 2001;79: 237–243.
2. Bourne RRA, Steinmetz JD, Flaxman S, Briant PS, Taylor HR, Resnikoff S. Trends in prevalence of blindness and distance and near vision impairment over 30 years: An analysis for the Global Burden of Disease Study. Lancet Glob Heal. 2021;9: e130–e143. doi:10.1016/S2214-109X(20)30425-3
3. Hashemi H, Fotouhi A, Yekta A, Pakzad R, Ostadimoghaddam H, Khabazkhoob M. Global and regional estimates of prevalence of refractive errors: Systematic review and meta-analysis. J Curr Ophthalmol. 2018;30: 3–22. doi:10.1016/j.joco.2017.08.009
4. Burton MJ, Ramke J, Marques AP, Bourne RRA, Congdon N. The Lancet Global Health Commission on Global Eye Health: vision beyond 2020. The Lancet Global Health.

Elsevier Ltd; 2021. pp. e489–e551. doi:10.1016/S2214-109X(20)30488-5

5. Narayan S. Addressing the Unmet Need: An Analysis of the Global Prevalence of Refractive Error and its Possible Solutions. Portland, OR; 2014 Jan. doi:10.15760/honors.87

6. Muma S, Naidoo KS, Hansraj R. Estimation of the Prevalence of Refractive Error in Kenya : A Systematic Review and Meta-Analysis. Optom Vis Perform. 2023;11.

7. Kandel H, Khadka J, Shrestha MK, Sharma S, Neupane Kandel S, Dhungana P, et al. Uncorrected and corrected refractive error experiences of Nepalese adults: a qualitative study. Ophthalmic Epidemiol. 2018;25: 147–161. doi:10.1080/09286586.2017.1376338

8. Eckert KA, Carter MJ, Lansingh VC, Wilson DA, Furtado JM, Frick KD, et al. A Simple Method for Estimating the Economic Cost of Productivity Loss Due to Blindness and Moderate to Severe Visual Impairment. Ophthalmic Epidemiol. 2015;22: 349–355. doi:10.3109/09286586.2015.1066394

9. Chakravarthy U, Biundo E, Saka RO, Fasser C, Bourne R, Little J-A. The Economic Impact of Blindness in Europe. Ophthalmic Epidemiol. 2017;24: 239–247. doi:10.1080/09286586.2017.1281426

10. Naidoo KS, Fricke TR, Frick KD, Jong M, Naduvilath TJ, Resnikoff S, et al. Potential Lost Productivity Resulting from the Global Burden of Myopia: Systematic Review, Meta-analysis, and Modeling. Ophthalmology. 2019;126: 338–346. doi:10.1016/j.ophtha.2018.10.029

11. Rein DB, Wittenborn JS, Zhang P, Sublett F, Lamuda PA, Lundeen EA, et al. The Economic Burden of Vision Loss and Blindness in the United States. Ophthalmology. 2022;129: 369–378. doi:10.1016/j.ophtha.2021.09.010

12. Marques AP, Ramke J, Cairns J, Butt T, Zhang JH, Muirhead D, et al. Global economic productivity losses from vision impairment and blindness. EClinicalMedicine. 2021;35: 100852. doi:10.1016/j.eclinm.2021.100852
13. Pezzullo L, Streatfeild J, Simkiss P, Shickle D. The economic impact of sight loss and blindness in the UK adult population. BMC Health Serv Res. 2018;18: 1–13. doi:10.1186/s12913-018-2836-0
14. International Agency for Prevention of. Ending avoidable sight loss. 2021. Available: https://www.iapb.org/wp-content/uploads/2022/02/2030inSight-Strategy-Document-Sep2021-English.pdf
15. Eckert KA, Carter MJ, Lansingh VC, Wilson DA. A Simple Method for Estimating the Economic Cost of Productivity Loss Due to Blindness and Moderate to Severe Visual Impairment. Ophthalmic Epidemiol. 2015;6586. doi:10.3109/09286586.2015.1066394
16. World Salaries. Average Caregiver Salary in Kenya for 2023. 2023 [cited 12 Aug 2023]. Available: https://worldsalaries.com/average-caregiver-salary-in-kenya/
17. Smith TST, Frick KD, Holden BA, Naidoo KS. Potential lost productivity resulting from the global burden of uncorrected refractive error. Bull World Health Organ. 2009; 431–437. doi:10.2471/BLT.08.055673
18. Fred Hollows Foundation T. Bringing light to sight in Kenya The benefits of eliminating avoidable. 2014. Available: https://www.hollows.org/us/where-we-work/africa/kenya-2
19. Trading Economics. Kenya - Employment In Services (% Of Total Employment) - 2023 Data 2024 Forecast 1991-2020 Historical. 2023 p. 1. Available: https://tradingeconomics.com/kenya/employment-in-services-percent-of-total-employment-wb-data.html

20. Brunes A, Heir T. Visual impairment and employment in Norway. BMC Public Health. 2022;22: 648. doi:10.1186/s12889-022-13077-0

21. World Bank. Employment rate in Kenya. 2023 [cited 21 Jun 2023]. Available: https://www.ceicdata.com/en/indicator/kenya/employed-persons

22. Junankar PN. Labor Force Participation BT - Economics of the Labour Market: Unemployment and the Costs of Unemployment. In: Junankar PNR, editor. Economics of The Labour Market. London: Palgrave Macmillan UK; 2016. pp. 38–41. doi:10.1057/9781137555199_5

23. CEIC. Kenya Labour Force Participation Rate, 1990 – 2023 | CEIC Data. 2023 [cited 8 Dec 2023]. Available: https://www.ceicdata.com/en/indicator/kenya/labour-force-participation-rate

24. Whitfield R, Schwab L, Ross-degnan D, Steinkuller P, Swartwood J. Blindness and eye disease in Kenya : ocular status survey results from the Kenya Rural Blindness Prevention Pro ject. Br J Ophthalmol. 1990; 333–340.

25. Fricke TR, Holden BA, Wilson DA, Schlenther G, Naidoo KS, Resnikoff S, et al. Global cost of correcting vision impairment from uncorrected refractive error. Bull World Health Organ. 2012; 728–738. doi:10.2471/BLT.12.104034

26. Kenya Ministry of Health. National Eye Health Strategic Plan. 2020. Available: https://www.medbox.org/document/national-eye-health-strategic-plan-2020-2025

27. Morjaria P, Ramson P. Services for refractive error in Kenya. J Ophthalmol East Cent South Africa. 2013. Available: https://www.researchgate.net/publication/279926992

28. Courtright P, Mathenge W, Kello AB, Cook C, Kalua K, Lewallen S. Setting targets for human resources for eye health in sub-Saharan Africa: what evidence should be used?

Hum Resour Health. 2016;14: 11. doi:10.1186/s12960-016-0107-x

29. Kenya Ministry of Health. Scope of practice for ophthalmic workers. 2022. Available: https://www.health.go.ke/

30. International Agency for Prevention and Blindness. Core competency for the eye health workforce in the WHO African region. 2016. Available: https://www.iapb.org/wp-content/uploads/Core-Competencies-for-Eye-HWF-in-WHO-AFRO-Region.pdf

31. Morjaria P, Ramson P, Gichangi M. Services for refractive error in Kenya. J Ophthalmol East Cent South Africa. 2013. Available: https://www.researchgate.net/publication/279926992

32. Morjaria P, Minto H, Ramson P, Gichangi M, Naidoo K, Gilbert C. Services for refractive error in Kenya : extent to which human resources and equipment are meeting VISION 2020 targets. J Ophthalmol East Cent South Africa. 2013;17: 44–49. Available: http://coecsa.org/ojs-2.4.2/index.php/JOECSA/article/view/78

33. Patel I, West SK. Presbyopia : prevalence , impact , and interventions. Community Eye Heal J. 2007;20: 51–52.

34. Eccles MP, Weijer C, Mittman B. Requirements for ethics committee review for studies submitted to Implementation Science. Implement Sci. 2011;6: 32. doi:10.1186/1748-5908-6-32

35. Leong SL, Teoh SL, Fun WH, Lee SWH. Task shifting in primary care to tackle healthcare worker shortages: An umbrella review. Eur J Gen Pract. 2021;27: 198–210. doi:10.1080/13814788.2021.1954616

36. Palmer J, Chinanayi F, Gilbert A, Pillay D, Fox S, Jaggernath J, et al. Mapping human resources for eye health in 21 countries of sub-Saharan Africa: current progress towards

VISION 2020. Hum Resour Health. 2014;12. doi:10.1186/1478-4491-12-44
37. Ramke J, Zwi AB, Silva JC, Mwangi N, Rono H, Gichangi M, et al. Evidence for national universal eye health plans. Bull World Health Organ. 2018;96: 695–704. doi:10.2471/BLT.18.213686
38. KK Optic. What is the Cost of Prescriptive Eyeglasses in Kenya? - KK Opticians. 2022. Available: https://kkopticals.co.ke/
39. Lapaire Glasses. Quality & Affordable Eyeglasses in Africa | Lapaire Glasses. 2018 [cited 7 May 2023]. Available: https://lapaire.africa/
40. Patel I, West SK. Presbyopia: prevalence, impact, and interventions. Community eye Heal. 2007;20: 40–41.
41. University of Nairobi. Department of Ophthalmology fee and funding. 2023 [cited 7 May 2023]. Available: https://opthalmology.uonbi.ac.ke/programs-content-type/master-medicine-ophthalmology
42. Masinde Muliro University. Bachelor of Optometry fee structure. 2019 p. 597360. Available: https://www.mmust.ac.ke/index.php/student-fee-structures
43. Kenya Medical Training College. Kenya Medical Training College | Training For Better Health. 2022. Available: https://kmtc.ac.ke/
44. Kenya National Bureau of Statistics. 2019 Kenya Population and Housing Census. 2019. Available: https://www.knbs.or.ke/2019-kenya-population-and-housing-census-reports/
45. International Agency for Prevention and Blindness. Strategy for the Elimination of Vision Impairment from Uncorrected Refractive Error. 2022. Available: https://www.iapb.org/learn/resources/strategy-for-the-elimination-of-vision-impairment-from-uncorrected-refractive-error/

46. Kenya Ministry of Health. National Strategic Plan For Eye Health and Blindness Prevention2012 to 2018. 2011.

47. Naidoo KS, Fricke TR, Frick KD, Jong M, Naduvilath TJ, Resnikoff S, et al. Potential Lost Productivity Resulting from the Global Burden of Myopia: Systematic Review, Meta-analysis, and Modeling. Ophthalmology. 2019;126: 338–346. doi:10.1016/j.ophtha.2018.10.029

48. Courtright P, Mathenge W, Kello AB, Cook C, Kalua K, Lewallen S. Setting targets for human resources for eye health in sub-Saharan Africa: What evidence should be used? Human Resources for Health. BioMed Central Ltd.; 2016. doi:10.1186/s12960-016-0107-x

49. Palmer JJ, Chinanayi F, Gilbert A, Pillay D, Fox S, Jaggernath J, et al. Mapping human resources for eye health in 21 countries of sub-Saharan Africa: Current progress towards VISION 2020. Hum Resour Health. 2014;12. doi:10.1186/1478-4491-12-44

50. Yorston D, Buchan J. Investment in human resources improves eye health for all. Community Eye Heal J. 2018;35: 37–39.

51. Khan AA, Khan NU, Bile KM, Awan H. Creating synergies for health systems strengthening through partnerships in Pakistan--a case study of the national eye health programme. East Mediterr Heal J. 2010;16 Suppl: S61-8.

52. Marques AP, Ramke J, Cairns J, Butt T, Zhang JH, Jones I. The economics of vision impairment and its leading causes: A systematic review. Clin Med (Northfield Il). 2022;46: 101354. doi:https://doi.org/10.1016/j.eclinm.2022.101354

53. World Bank. Macro Poverty Outlook for Kenya. 2022 pp. 1–2. Available: https://www.worldbank.org/en/publication/macro-poverty-outlook

54. World Health Organization. World report on vision. 2019. doi:http://apps.who.int/iris.

55. Kenya Laws. The constitution of Kenya, 2010. 2010. Available: http://kenyalaw.org/kl/index.php?id=398

56. Luke B, Chu V. Social enterprise versus social entrepreneurship : An examination of the ' why ' and ' how ' in pursuing social change. Int Small Bus J. 2013;31: 765–766. doi:10.1177/0266242612462598

57. Nyawira L, Tsofa B, Musiega A, Munywoki J, Njuguna RG, Hanson K, et al. Management of human resources for health: implications for health systems efficiency in Kenya. BMC Health Serv Res. 2022;22: 1046. doi:10.1186/s12913-022-08432-1

58. Haleem A, Javaid M, Singh RP, Suman R. Telemedicine for Healthcare : Capabilities , features , barriers , and applications Telemedicine for healthcare : Capabilities , features , barriers , and applications. Sensors Int. 2021;2: 100117. doi:10.1016/j.sintl.2021.100117

59. Kim T, Zuckerman JE. Realizing the potential of telemedicine in global health. J Glob Health. 2019;9. doi:10.7189/jogh.09.020307

60. Chia MA, Turner AW. Benefits of Integrating Telemedicine and Artificial Intelligence Into Outreach Eye Care: Stepwise Approach and Future Directions. Front Med. 2022;9: 1–6. doi:10.3389/fmed.2022.835804

61. Randhawa H, Morettin C, McLeod H, Wyles E, Sanghera N, Kattouf V, et al. The validity of spectacle prescriptions via tele-optometric comprehensive eye examinations; a pilot study. Invest Ophthalmol Vis Sci. 2020;61: 1604.

62. Ulldemolins AR, Lansingh VC, Valencia LG, Carter MJ, Eckert KA. Review Article Social inequalities in blindness and visual impairment : A review of social determinants. 2011. doi:10.4103/0301-4738.100529

63. Zhang JH, Ramke J, Jan C, Bascaran C, Mwangi N, Furtado JM, et al. Review Advancing the Sustainable Development Goals through improving eye health : a scoping review. Lancet Planet Heal. 2022; 270–280. doi:10.1016/S2542-5196(21)00351-X

Click here to access/download
Supporting Information
Data for the study.pdf

Estimation of the lost productivity to the GDP and the national cost of correcting visual impairment from refractive error in Kenya

Shadrack Muma[1¶], Kovin Shunmugam Naidoo[1, 2¶], Rekha Hansraj[1¶]

[1]Department of Optometry, College of Health Sciences, University of KwaZulu-Natal, Durban, South Africa

[2]OneSight EssilorLuxottica Foundation, Paris, France

***** Corresponding author

Email- mumashadrack275@gmail.com (SM)

[¶]These authors contributed equally to this work.

Abstract

Background: In developing countries such as Kenya, minimal attention has been directed towards population based studies on uncorrected refractive error (URE). However, in the absence of population based studies, other avenues are desirable to showcase to stakeholders in eye health the worth of addressing URE. Hence this study estimated the lost productivity to the Gross Domestic Product (GDP) as a result of URE and the national cost required to address visual impairment from URE in Kenya.

Methods: The lost productivity to the GDP for the population aged 16-60 years was calculated. Thereafter the productivity loss of the caregivers of severe visual impaired individuals was computed as a product of the average annual productivity for each caregiver and a 5% productivity loss due to visual impairment. The productivity benefit of correcting refractive error was estimated based on the minimum wage for individuals aged 16-60 years with URE. Estimation of the national cost of addressing URE was based on spectacle provision cost, cost of training functional clinical refractionists and the cost of establishing vision centres. A cost benefit analysis was undertaken based on the national cost estimates and a factor of 3.5 times.

Results: The estimated lost productivity to the GDP due to URE in in Kenya is approximately US$ 671,455,575 -US$ 1,044,486,450 annually for population aged 16-60 years. The productivity loss of caregivers for the severe visually impaired is approximately US$ 13,882,899 annually. Approximately US$ 246,750,000 is required to provide corrective devices, US$ 413,280- US$ 108,262,300 to train clinical refractionists and US$ 39,800,000 to establish vision centres. The productivity benefit of correcting visual impairment is approximately US$ 41,126,400 annually. Finally, a cost benefit analysis showed a return of US$ 378,918,050 for human resource, US$ 863,625,000 for corrective devices and US$ 139,300,000 for establishment of vision centres.

Conclusion: The magnitude of productivity loss due to URE in Kenya is significant warranting prioritization of refractive error services by the government and all stakeholders since any investment directed towards addressing URE has the potential to yield a positive return.

Keywords: Productivity loss; gross domestic product; telemedicine; uncorrected refractive error, national costs; cost benefit analysis; productivity benefit

Introduction

Refractive error which is a type of vision problem that makes it hard to see clearly is the leading cause of visual impairment (VI) globally [1]. According to the Global Burden of Disease 2020, 338.8 million people globally had moderate and severe visual impairment (MSVI) with a projection of 535 million by 2050 if no interventions are taken to reduce avoidable blindness [2]. As a result, all stakeholders in eye health should direct efforts towards addressing URE for net economic gain [3–5]. Holistically, eye care professionals should be at the forefront in providing evidence on URE and showcase to the government in particular, why policy changes and actions should be undertaken to address URE. In Kenya it is estimated that approximately 3.5 million individuals suffers from RE warranting the need for actions to address this condition [6]. To achieve this in developing countries such as Kenya, eye care professionals in collaboration with other stakeholders such as social entrepreneurs, Ministry of Health and commercial entrepreneurs should support population based studies. The population based studies would showcase the actual burden and influence the policy makers to establish policies and recognize the importance of addressing the burden attributed to URE [3]. Even though evidence shows that URE impacts negatively on the quality of life [7], standard operating procedures and central monitoring should be in place to showcase to the government the burden attributed to URE from population based studies so as to influence the allocation of resources to address this condition in Kenya. The financial impact of vision loss on the global economy has been a major concern over the past decade [8–13]. As a result, showcasing the financial impact attributed to URE is desirable to influence the policy makers on the need for an action. Therefore, this study intended to establish the lost productivity to the GDP as a result of URE and the national cost required to address visual impairment (VI) from URE in Kenya. It is anticipated that this estimate can act as a baseline and showcase to the government, eye care professionals and all stakeholders in eye

health the worth of directing efforts towards addressing accessibility, availability and affordability of refractive error (RE) services to achieve the 2030 IN SIGHT [14]

Materials and methods

According to Eckert and colleagues [15], MSVI results in a range of reduced productivity with 30%-55% not working while 45%-70% have their earning reduced by 35%. In Kenya, the minimum wage (MW) which is the lowest remuneration that employers can legally pay their employee is approximately US$ 101.505 per month [16]. The annual MW for the 45%-70% of the individuals with URE who are working was computed. Thereafter, a factor of 0.35 was used to estimate the reduced earnings for the visually impaired individuals who are working. Currently in Kenya, approximately 3.5 million people are estimated to suffer from RE [6]. The method proposed by Eckert and colleagues [15] in calculating the lost productivity as a result of MSVI was adopted. The productivity loss was computed as a product of (0.45 of the affected population * 0.35 of reduced earnings) and (0.7 of the affected population * 0.35 of reduced earnings) [15]. The population aged 16-60 years with VI was included for the estimations with the assumption that the population below 16 years and those above 60 years with URE yield no economic productivity to a country's GDP as proposed by Smith and colleagues [17].

The productivity benefit of addressing URE among visually impaired individuals in Kenya of working age (16-60 years) was estimated. In Kenya, approximately 51% of the visually impaired individuals constitute the working age group [18] with an ER of 16% [19]. In the absence of VI, those treated would be employed at the same rate as the individuals with good vision [20]. In Kenya, the employment rate is estimated at 38.5% nationally for the population aged 16-60 years with a MW of US$ 101.505 monthly [19]. To scale the current ER for visually impaired individuals to the national average of 38.5%, additional investments beyond the recurrent

expenditure in eye health care to develop sustainable primary and secondary healthcare systems are desirable. An assumption was made that if individuals with VI benefits from spectacles and low vision devices, then the ER would potentially be scaled to the national average of 38.5% [19]. An assumption was also made that if the visually impaired individuals who have attained the working age are provided with corrective devices and employed thereafter then the productivity benefit would increase. The productivity benefit was computed in three perspectives. Firstly, the productivity benefit for the 51% of the individuals who are visually impaired and have attained the employment age was computed as a product of the annual MW and the population [21]. The unadjusted productivity benefit was adjusted using the LFPR which is the estimate of the economy's active workforce [22]. In Kenya, the LFPR is approximately 0.739 [23]. Secondly, the current productivity benefit accrued from the 16% of the visually impaired in Kenya was calculated. Finally, a productivity benefit was computed with an assumption that the ER which is the measure of the extent to which available labour resources are being utilized for visually impaired is scaled to the national average of 38.5% [22].

The productivity loss of the caregivers for severe visually impaired individuals to the GDP was computed. The population aged 16-60 years with normal vision was included with the assumption that they are productive and can take care of visually impaired individuals. The average income of the normal sighted caregivers aged 16-60 years annually was calculated. Given that approximately 3.5 million Kenyans are visually impaired–[6], an assumption was made that the population with severe VI would require some sought of care in the absence of corrective devices. Out of the 3.5 million Kenyans with MSVI, it is estimated that approximately 7.5% have severe VI [24]. Therefore, it was assumed that there is one caregiver for every individual with severe VI. The number of required caregivers was estimated based on the

number of individuals with severe VI in Kenya. Based on a study by Fricke and colleagues [25], it was assumed that every caregiver for a visually impaired individual experiences a productivity loss of 5% on the total average yearly productivity. Hence the productivity loss of caregivers was computed as a product of the average annual productivity for each caregiver and the 5% productivity loss due to the care provided. It was assumed that if severe VI is addressed, it is expected that caregivers will be able to increase their productivity through employment, education and other approaches.

The national cost calculation entailed summation of the cost required to establish vision centres (VCs), the cost required to scale functional clinical refractionists and the cost required to provide spectacles based on the estimated prevalence of URE in Kenya. An assumption was made that URE cannot be addressed effectively in the absence of adequate VCs where patients with RE can access RE services. The cost required to establish the VCs in Kenya was computed based on estimates from the IAPB of US$ 50,000 [14] and the Ministry of Health of Kenya of US$ 100,000 [26] per vision centre (VC). The number of required VCs was estimated based on the population density and the Ministry of Health in Kenya consideration in establishing a healthcare facility [26]. In Kenya, the Ministry of Health recommends establishment of health facilities in an area with a population density of 100,000 [26]. However, the services offered in such healthcare facilities may not be inclusive of RE services. Therefore, this paper recommended integration of VCs within the existing healthcare facilities to offer RE services to a population of 100,000. This paper also recommended that in the presence of resources, one VC should serve a population of 50,000 with an assumption that this will enhance quality RE services delivery. Given the limited resources in Kenya to scale the VCs [27], This paper proposed a holistic conservative recommendation of one VC per 100,000 populations based on the Ministry of

Health criteria for establishing healthcare facility [26]. The study also recommended that telemedicine integration into the eye health ecosystem should be adopted to ensure that the limited human resource available in Kenya could scale service delivery to the VCs. The proposed telemedicine should entail a communication among eye care professionals in regards to a patient presentation and course of management.

The national cost estimate required to scale human resource was undertaken. The calculations conducted were based on the Ministry of Health recommendations on the ratios of human resource to population in Kenya [26]. The recommendations by the Ministry of Health on eye care professionals to population includes; ophthalmologists 1:250,000, optometrists 1:250,000, ophthalmic clinical officers 1:100,000, optometry technologists 1:100,000, ophthalmic clinical officers/cataract surgeons 1:125,000 and ophthalmic nurses 1: 100,000 [28]. Even though the scope of practice for ophthalmic workers in Kenya allows the above categories of eye care professionals to undertake refraction, not all meets the threshold of a functional clinical refractionist who undertakes refraction 100% of their time [29]. The World Health Organization (WHO) recommendations on the minimum eye care teams for one per million population includes ophthalmologists=4, optometrists=10, primary healthcare workers=200 and allied health professionals=10 [30]. The key activities proposed by the WHO recognize the potential of optometrists in meeting the threshold for a functional clinical refractionist [30]. Based on the range of services that ophthalmic workers in Kenya undertakes as per their scope of practice [29], this paper estimated that ophthalmologists may spend roughly 20% of their time doing refraction, ophthalmic clinical officers roughly 30% of their time, optometrists may spend approximately 90% of their time and finally ophthalmic nurses may spend roughly 15% of their time [29]. This estimation was intended to provide a justifiable ground for stakeholders in eye

health to direct efforts towards scaling eye care professionals meeting the threshold for functional clinical refractionists in Kenya. The number of required functional clinical refractionists from the existing eye care professionals having the potential to conduct refraction in Kenya was calculated based on the estimates by Morjaria and collegues [32]. The deficit of functional clinical refractionists was computed based on the WHO recommendation of one functional clinical refractionist per 100,000 populations [30]. Even though the WHO recommendation is ideal, this paper presents different proportions that could be achieved through integration of technological approaches such as telemedicine. As a result, this study estimated the number of functional clinical refractionists that should be trained to undertake refraction if adequate resources are available and telemedicine integration is prioritized. The calculations were based on the ratio of one refractionist per 50,000, 25,000 and 10,000 population. This study recommended that if one refractionist per 50,000, 25,000 and 10,000 population ratios are to be adopted then strengthened telemedicine integration is desirable. The estimated cost for training the deficit of functional clinical refractionists was computed based on the existing each eye care professional in Kenya who operates as functional clinical refractionists. The cost required to train functional clinical refractionists was computed for each category of the eye care professional based on the deficit and the recommendations by the WHO, Ministry of Health and this study recommendations based on telemedicine integration.

Finally, based on the national cost required to provide the 3.5 million individuals with URE in Kenya with spectacles-[6], a review of the current private sector rates, as well as those of social enterprises (SE) was undertaken with an aim of estimating the cost required to provide RE services for the population in dire need of these in Kenya. The public health sector in Kenya was excluded as they do not offer spectacles but instead referring patients to the private sector for

spectacles. The SE located within the public sector such as the Kenya Society for the Blind were included. To estimate the number of individuals with URE who already have corrective devices, the 6% (210,000) estimates of the number of spectacles wearers in Tanzania was adopted [33]. The estimated number of individual with URE already having corrective devices was excluded from the national cost estimates. The national cost for correcting URE for the 3.5 million Kenyans was computed based on the average spectacles charges from the private sector and the SE sectors.

After the national cost estimates for addressing URE in Kenya were provided, a cost benefit analysis was conducted to show whether it would be worth investing the total amount required to provide corrective devices, scaling human resources and establishing VCs in Kenya. Considering that the benefit of addressing URE remains significant and mostly overrides cost invested in addressing URE by a factor of 3.5 times [18], the implication is that if one dollar is invested in addressing URE then a possible return of US$ 3.56 will be accrued. Hence the cost benefit analysis was computed as a product of the cumulative national cost required to address URE and the factor of 3.5 times was used. The estimate was intended to showcase whether it is worth investing the national cost of addressing URE by stakeholders in eye health in Kenya.

This study was approved by Maseno University Ethics Review Committee in Kenya reference MUERC/1051/22 and the Biomedical Research Ethics Committee in South Africa reference BREC/00004105/2022. The ethics review committee waived the requirements for the informed consent. This waiver was attributed to the nature of data recorded which is available within the public domains with no human subject involved [34].

Results

Lost productivity of visually impaired individuals to the GDP

The annual MW for the 45%-70% of the working individuals with MSVI in Kenya is approximately US$ 1,918,444,500 –US$ 2,984,247,000. The estimated productivity loss to the GDP as a result of VI in Kenya is approximately US$ 671,455,575 -US$ 1,044,486,450 annually.

Lost productivity by caregivers of visually impaired individuals to the GDP

The number of caregivers required to attend to the 7.5% Kenyans with severe VI is approximately 227,951 caregivers. The average income for a caregiver of the visually impaired person in Kenya is approximately US$ 101.505 monthly [16]. Hence the annual MW for the estimated 227,951 caregivers is approximately US$ 277,657,995. Therefore, if the 5% productivity loss as a result of taking care of the severe visually impaired individuals is adopted, then approximately US$ 60.90 per caregiver is lost annually. Hence cumulatively a total of US$ 13,882,899 is lost annually if the 7.5% of severe visually impaired individual receive care from the healthy population.

National cost estimates for addressing uncorrected refractive error in Kenya

Based on the scope of practice for ophthalmic workers allowed to undertake refraction in Kenya [29], the estimates on the proportions of professionals who undertake refraction by Morjaria and colleagues [31] in Kenya and this study assumption is that ophthalmologists, ophthalmic clinical officers, optometrists and ophthalmic nurses may spend approximately 20%, 30%, 90% and 15% of their time respectively doing refraction [29]. This study estimated that approximately 560 functional clinical refractionists exist in Kenya. Based on this study recommendation of one functional clinical refractionist per 10,000 populations if telemedicine is integrated, 4,756 functional clinical refractionists will be required in Kenya as shown in Table 1.

Table 1: Deficit of functional clinical refractionists required in Kenya based on different

practitioner – population ratios

Recommendations	Source	Required functional clinical refractionists	Existing functional clinical refractionists	Deficit
1:250,000	[35]	190	560	Exceeds
1:100,000	[29]	475	560	Exceeds
1:50,000	[36]	951	560	391
1:25,000	[37]	1,942	560	1,382
1:10,000	[28]	4,756	560	4,196

The current spectacle charges from the private sector in Kenya is estimated at an average cost of US$ 75 [38] and that of SE an average of US$ 35 [39]. Hence, with the current rates, majority of the Kenyan population are unable to afford the spectacles as only 38.5% are employed [19]. In consideration of the estimated prevalence of URE in Kenya of 3.5 million–[6], the estimated national cost for addressing URE using the private sector rate of US$ 75 is approximately US$ 262,500,00 while the estimated national cost based on SE rates of US$ 35 is approximately US$ 122,500,00. With the assumption that there is a population already having corrective devices, this study adopted the estimates from Tanzania in which 6% (210,000) of the population with URE are estimated to have spectacles already-[40]. The population with RE who already have corrective devices was excluded from the estimates. Out of the 3.5 million individuals with RE in Kenya, approximately 210, 000 already have corrective devices. Therefore, the estimated national cost for addressing URE based on the private sector rate was approximately US$ 246,750,000 while the estimated national cost based on SE rates was approximately US$ 115,150,000.

National cost of training human resource to address uncorrected refractive error in Kenya

Currently, the cost of training an eye care professional in Kenya is approximately US$ 1,680 -

US$ 20,450 [41]. Hence if human resource is to be scaled based on various ratios recommended by the WHO, the Ministry of Health and this study recommendations, then approximately 540 functional clinical refractionists will be required for 1:100,000, 1,080 functional clinical refractionists for 1:50,000, 2,160 functional clinical refractionists for 1:25,000 and 5,400 functional clinical refractionists for 1:10,000. The cost required to train the professionals based on the ratios above will be approximately US$ 413,280- US$ 108,262,300. Details are shown in Table 24.

Table 24: Estimated cost of training human resource in eye health in Kenya based on professionals and proposed ratios

				**Total cost (US$) for training functional clinical refractionist; n=required number**			
**Professionals**	**Cost (US$) of training to completion**	**Source**	**Existing functional clinical refractionists**	**Required (n=540) 1:100,000** [29]	**Required (n=1,080) 1:50,000** [36]	**Required (n=2,160) 1:25,000** [37]	**Required (n=5,400) 1:10,000** [28]
Optometrist	7,000	[42]	178	2,534,000	6,314,000	13,874,000	36,554,000
Ophthalmologist	20,450	[41]	106	8,875,300	19,918,300	42,004,300	108,262,300
Ophthalmic Clinical Officer/CS	3,004	[43]	164	1,129,504	2,751,664	5,995,984	15,728,944
Optometry technologist	1,680	[43]	294	413,280	1,320,480	3,134,880	8,578,080
Ophthalmic nurse	3,004	[43]	80	1,381	3,004,000	6,248,320	15,981,280

Conventionally, if one VC is established to serve a population of 50,000, then approximately 1,080 functional VCs will be required to serve the Kenyan population conveniently given that currently the Kenyan population is approximately 54 million [44]. Using the International Agency for Prevention and Blindness (IAPB) recommendation of an average of US$50,000 for equipping and establishing a VC [45] and the Kenya Ministry of Health, [46] estimate of

US$100,000, the estimated cost required to establish the VCs in Kenya are shown in Table 3.

Table 3: Estimated cost of establishing the required VCs in Kenya

VCs per population	Required VCs	Existing VCs	Deficit VCs	Cost of establishing the VCs	
				IAPB Recommended rates (US$)	Ministry of Health, Kenya rates (US$)
1:100,000	475	77	398	19,900,000	39,800,000
1:50,000	951	77	874	43,700,000	87,400,000

Productivity benefit to correcting the visually impaired individuals in Kenya

To scale the ER for visually impaired to the national average of 38.5%, additional 22.5% of the visually impaired should be employed. Out of the 3.5 million individuals in Kenya with VI [6], 1,785,000 have attained the employment age. Considering the MW of US$ 101.505 monthly [21], the unadjusted productivity benefit will be approximately US$ 2,174,237,100 annually if the 51% of the visually impaired individuals within 16-60 years are employed. This estimate was considered exceptional given that not all visually impaired individuals may meet the thresholds and requirements to be employed. As a result, the unadjusted productivity benefit was adjusted using the LFPR of 0.739. The adjusted productivity benefit is approximately US$ 1,606,761,216 annually. The second productivity benefit currently generated by the 16% of the visually impaired in Kenya is approximately US$ 41,126,400 annually. Finally, it was assumed that if the ER for visually impaired individuals is scaled to the national average of 38.5% then the productivity benefit will be approximately US$ 98,960,400 annually based on the current dollar exchange rates.

Cost benefit analysis

The estimation of the cost benefit analysis was based on three aspects. The first aspect was the

human resource. Based on this aspect, a cost benefit analysis was undertaken to show whether the cost of US$ 108,262,300 required in scaling the human resource would yield a positive return worth the investment. The number of the required human resources was determined based on the Ministry of Health recommendation of 1:100,000 and this study recommendation of 1:50,000, 1:25,000 and 1:10,000 for professional to the population. The second aspect was provision of corrective devices. This study computed a cost benefit analysis to show whether investing the cost of US$ 246,750,000 in providing corrective devices to the 3.5 million individuals with RE in Kenya was worth. The final aspect was the VCs establishment. This study determined the cost benefit analysis to show whether it is worth investing US$ 39,800,000 to establish VCs in Kenya. For each US$1 invested in the efforts to eliminate VI in Kenya, a return of US$3.56 could potentially be realized [18]. The cost benefit analysis estimate was computed as a product of the national cost estimates for scaling human resource, establishing VCs and providing corrective devices and the factor of 3.5 times. The result is shown in Table 4.

Table 4: Cost benefit analysis for investments towards addressing uncorrected refractive error in Kenya

Area of investment	National cost estimate required (US$)	Factor estimate	Return on investment (US$)
Human resources	108,262,300	3.5	378,918,050
Corrective devices	246,750,000	3.5	863,625,000
Vision centres	39,800,000	3.5	139,300,000

Discussion

The growth of an economy's GDP could be determined by the population aged 16-60 years having the potential to undertake activities inclined towards enhancing net economic gain [47]. However, this can only be achieved if the population with disability is provided with the right devices to address their condition which limits them from undertaking their daily activities. Through the conservative approach, this study estimates a lost productivity of approximately US$ 671,455,575 -US$ 1,044,486,450 annually to the GDP as a result of URE. Similarly, for caregivers, the lost productivity to the GDP is estimated at approximately US$ 13,882,899 annually. This justifies the assertion that URE contributes to the country GDP loss warranting the government to direct more efforts towards addressing URE. With the limited resources [48–50] during the COVID-19 pandemic, the government allocated resources to address the pandemic, a clear indication that evidence on the severity of a condition may force the government to take action and address the situation. Hence the burden of URE in Kenya should be showcased to the government by eye care professionals and other stakeholders to justify why attention should be directed towards addressing URE. However, there is lack of standard operating procedures and central monitoring as a minimum warranting the need for collaboration between the eye care professionals, researchers and all stakeholders in eye health. As it stands, the government and other stakeholders in eye health are directing efforts towards addressing URE based on global assumptions and estimates since a clear picture of the burden of URE in developing countries such as Kenya remains unknown. Therefore, the estimates provided in this paper are intended to act as a baseline to showcase to stakeholders in eye health the need for action to address URE.

Uncorrected refractive error contributes negatively to the GDP [51]. However, dispensing a simple pair of spectacles not only impacts on the quality of life of an individual, but contributes

to net economic gain [52]. This study shows that the productivity benefit of correcting VI in Kenya is approximately US$ 41,126,400 annually. This justifies the value of providing spectacles and other corrective devices to patients with RE. However, with approximately 59.6% to 85.7% of Kenyans living on less than US$ 1.9 a day [53], it would be necessary for the government to integrate spectacles and other corrective devices within the public sector so as to assist patients with URE to gain access and be able to afford the available RE services. More holistically, designing an approach where corrective devices are provided at prices based on the average income of everyone across the economic pyramid is desirable. This is to ensure that patients with RE become productive and contribute to the GDP since any investment on URE contributes a return of a factor of 3.5 times [18].

Uncorrected RE remains the leading cause of VI globally [54] and Kenya is not an exception [6]. As a result, eye care professionals in collaboration with stakeholders in eye health should be at the forefront in advocating for the inclusion of eye health within the public sector. Although the constitution of Kenya 2010 stipulates that the government should ensure access to quality healthcare [55], it is incapacitated with the private sector dominating the eye health sector. With the current charges for spectacles in the private sector [38], the majority of the population across the economic pyramid cannot afford the spectacles primarily, as the poverty rate ranges between 59.6%-85.7% with the majority living on less than US$ 1.9 a day [53]. Therefore, achieving an effective RE coverage will require partly the government to recognize the efforts that various stakeholders are making towards addressing URE. Tactically, considering the relatively reasonable charges by the SE, the government should establish policies to allow the SE, which are organizations that participate in business ventures through a commercial approach in order to fulfill a social purpose [56], to introduce RE services within the existing public sector institutions

so as to scale effective RE coverage in a more cost effective and sustainable way.

Human resource development is a fundamental concept in a health system and capacity building strategies should be built on the community requirements [57]. While the human resources remains a major challenge within the eye care ecosystem in developing countries such as Kenya [36], a needs based approach of training professionals together with integrated telemedicine which is the mode of healthcare delivery through information and technology, is desirable [58,59]. Telemedicine has been applied within remote settings to overcome geographical barriers to healthcare access, providing an alternative means of connecting patients to specialists [60]. A meeting abstract by Randhawa and colleagues [61] examining telehealth delivered subjective refraction found no statistically significant difference between in person and telehealth delivered subjective refraction. This study has shown that that there are approximately 822 eye care providers who undertake refraction in Kenya. However, not all of them meet the threshold of functional clinical refractionists who undertake refraction one hundred percent of their time. As a result, out of the 822 eye care providers who undertake refraction, approximately 560 meet the threshold of functional clinical refractionists while others engage on various roles such as screening for eye diseases, eye surgery, training and treatment [29]. Therefore, if the recommendation of 1:50,000 is adopted as suggested by Fricke and colleagues [25] in Kenya, then an additional 391 functional clinical refractionists are required. However, this study estimates that for effective RE coverage, a conventional recommendation of 1:10,000 is desirable and if adopted, 4840 clinical refractionists should be trained at a cost of approximately US$ 108,262,300. Therefore, considering the limited resources and the high cost of training for eye care professionals who end up not operating as functional clinical refractionists, needs based training should be used and focus on optometrists and integrating the telemedicine to scale effective RE

coverage in Kenya.

In Kenya, there are approximately 12,393 health facilities, with only 77 having functional optical units [31]. This low number of units in Kenya is linked to the lack of a demonstrated national prevalence of URE from population based studies which can showcase to the government the burden of URE and the need for action in this regard. However, for effective RE coverage to be achieved, the establishment of VCs are desirable. This study estimates that approximately 951 VCs are required for effective RE service delivery. This implies that one VC is desirable per 50,000. With limited resources in Kenya, a holistic approach of one VC per 100,000 is desirable warranting 475 of such units to be established in Kenya at a cost of US$ 39,800,000 based on the Ministry of Health of Kenya estimate of funding per VC of US$100,000 [26]. The establishment of the VCs is costly; however, a cost benefit analysis still shows that a return will be accrued if such units are established. Therefore, a technological approach like telemedicine should be integrated within the established optical units to scale RE service delivery.

Addressing URE should conventionally be prioritized for quality life. Hence considering the current situation in which approximately 83% of VI is present in low-income and middle-income countries among the underserved population [62,63], the productivity loss might be huge given the limited estimates showing the cost benefit analysis of URE. As a result, this paper will act as baseline for stakeholders in eye health especially the government to understand the value of investing the estimated national cost for addressing URE in Kenya. Addressing URE will not only be beneficial to the individuals but will also benefit the caregivers of individuals with URE and contribute to the economic growth of a country. This study has showed that any investment directed towards addressing URE contribute to a positive return. As a result, it is worthy of attention for stakeholders in eye health sector to invest more in addressing URE as a projected

positive return will be accrued provided the investment is properly executed by professionals.

In conclusion, URE contributes to lost productivity and reduction of the GDP in Kenya, therefore investing the national cost in training human resources, establishing VCs and providing spectacles to the population in dire need will contribute a productivity benefit. Although the estimates in this study may not be exact due to aspects like varying exchange rates for a dollar, to the best of our knowledge, this is the first study to be conducted in Kenya on the lost productivity to the GDP as a result of URE. It also provides an overview of the current situation in Kenya highlighting why investments towards addressing URE should be prioritized by the government and stakeholders in eye health.

Acknowledgement

The authors would like to acknowledge the statistician for assisting during data analysis.

References

1. Dandona R, Dandona L. Refractive error blindness. Bull World Health Organ. 2001;79: 237–243.
2. Bourne RRA, Steinmetz JD, Flaxman S, Briant PS, Taylor HR, Resnikoff S. Trends in prevalence of blindness and distance and near vision impairment over 30 years: An analysis for the Global Burden of Disease Study. Lancet Glob Heal. 2021;9: e130–e143. doi:10.1016/S2214-109X(20)30425-3
3. Hashemi H, Fotouhi A, Yekta A, Pakzad R, Ostadimoghaddam H, Khabazkhoob M. Global and regional estimates of prevalence of refractive errors: Systematic review and meta-analysis. J Curr Ophthalmol. 2018;30: 3–22. doi:10.1016/j.joco.2017.08.009
4. Burton MJ, Ramke J, Marques AP, Bourne RRA, Congdon N. The Lancet Global Health Commission on Global Eye Health: vision beyond 2020. The Lancet Global Health.

Elsevier Ltd; 2021. pp. e489–e551. doi:10.1016/S2214-109X(20)30488-5

5. Narayan S. Addressing the Unmet Need: An Analysis of the Global Prevalence of Refractive Error and its Possible Solutions. Portland, OR; 2014 Jan. doi:10.15760/honors.87

6. Muma S, Naidoo KS, Hansraj R. Estimation of the Prevalence of Refractive Error in Kenya : A Systematic Review and Meta-Analysis. Optom Vis Perform. 2023;11.

7. Kandel H, Khadka J, Shrestha MK, Sharma S, Neupane Kandel S, Dhungana P, et al. Uncorrected and corrected refractive error experiences of Nepalese adults: a qualitative study. Ophthalmic Epidemiol. 2018;25: 147–161. doi:10.1080/09286586.2017.1376338

8. Eckert KA, Carter MJ, Lansingh VC, Wilson DA, Furtado JM, Frick KD, et al. A Simple Method for Estimating the Economic Cost of Productivity Loss Due to Blindness and Moderate to Severe Visual Impairment. Ophthalmic Epidemiol. 2015;22: 349–355. doi:10.3109/09286586.2015.1066394

9. Chakravarthy U, Biundo E, Saka RO, Fasser C, Bourne R, Little J-A. The Economic Impact of Blindness in Europe. Ophthalmic Epidemiol. 2017;24: 239–247. doi:10.1080/09286586.2017.1281426

10. Naidoo KS, Fricke TR, Frick KD, Jong M, Naduvilath TJ, Resnikoff S, et al. Potential Lost Productivity Resulting from the Global Burden of Myopia: Systematic Review, Meta-analysis, and Modeling. Ophthalmology. 2019;126: 338–346. doi:10.1016/j.ophtha.2018.10.029

11. Rein DB, Wittenborn JS, Zhang P, Sublett F, Lamuda PA, Lundeen EA, et al. The Economic Burden of Vision Loss and Blindness in the United States. Ophthalmology. 2022;129: 369–378. doi:10.1016/j.ophtha.2021.09.010

12. Marques AP, Ramke J, Cairns J, Butt T, Zhang JH, Muirhead D, et al. Global economic productivity losses from vision impairment and blindness. EClinicalMedicine. 2021;35: 100852. doi:10.1016/j.eclinm.2021.100852
13. Pezzullo L, Streatfeild J, Simkiss P, Shickle D. The economic impact of sight loss and blindness in the UK adult population. BMC Health Serv Res. 2018;18: 1–13. doi:10.1186/s12913-018-2836-0
14. International Agency for Prevention of. Ending avoidable sight loss. 2021. Available: https://www.iapb.org/wp-content/uploads/2022/02/2030inSight-Strategy-Document-Sep2021-English.pdf
15. Eckert KA, Carter MJ, Lansingh VC, Wilson DA. A Simple Method for Estimating the Economic Cost of Productivity Loss Due to Blindness and Moderate to Severe Visual Impairment. Ophthalmic Epidemiol. 2015;6586. doi:10.3109/09286586.2015.1066394
16. World Salaries. Average Caregiver Salary in Kenya for 2023. 2023 [cited 12 Aug 2023]. Available: https://worldsalaries.com/average-caregiver-salary-in-kenya/
17. Smith TST, Frick KD, Holden BA, Naidoo KS. Potential lost productivity resulting from the global burden of uncorrected refractive error. Bull World Health Organ. 2009; 431–437. doi:10.2471/BLT.08.055673
18. Fred Hollows Foundation T. Bringing light to sight in Kenya The benefits of eliminating avoidable. 2014. Available: https://www.hollows.org/us/where-we-work/africa/kenya-2
19. Trading Economics. Kenya - Employment In Services (% Of Total Employment) - 2023 Data 2024 Forecast 1991-2020 Historical. 2023 p. 1. Available: https://tradingeconomics.com/kenya/employment-in-services-percent-of-total-employment-wb-data.html

20. Brunes A, Heir T. Visual impairment and employment in Norway. BMC Public Health. 2022;22: 648. doi:10.1186/s12889-022-13077-0

21. World Bank. Employment rate in Kenya. 2023 [cited 21 Jun 2023]. Available: https://www.ceicdata.com/en/indicator/kenya/employed-persons

22. Junankar PN. Labor Force Participation BT - Economics of the Labour Market: Unemployment and the Costs of Unemployment. In: Junankar PNR, editor. Economics of The Labour Market. London: Palgrave Macmillan UK; 2016. pp. 38–41. doi:10.1057/9781137555199_5

23. CEIC. Kenya Labour Force Participation Rate, 1990 – 2023 | CEIC Data. 2023 [cited 8 Dec 2023]. Available: https://www.ceicdata.com/en/indicator/kenya/labour-force-participation-rate

24. Whitfield R, Schwab L, Ross-degnan D, Steinkuller P, Swartwood J. Blindness and eye disease in Kenya : ocular status survey results from the Kenya Rural Blindness Prevention Pro ject. Br J Ophthalmol. 1990; 333–340.

25. Fricke TR, Holden BA, Wilson DA, Schlenther G, Naidoo KS, Resnikoff S, et al. Global cost of correcting vision impairment from uncorrected refractive error. Bull World Health Organ. 2012; 728–738. doi:10.2471/BLT.12.104034

26. Kenya Ministry of Health. National Eye Health Strategic Plan. 2020. Available: https://www.medbox.org/document/national-eye-health-strategic-plan-2020-2025

27. Morjaria P, Ramson P. Services for refractive error in Kenya. J Ophthalmol East Cent South Africa. 2013. Available: https://www.researchgate.net/publication/279926992

28. Courtright P, Mathenge W, Kello AB, Cook C, Kalua K, Lewallen S. Setting targets for human resources for eye health in sub-Saharan Africa: what evidence should be used?

Hum Resour Health. 2016;14: 11. doi:10.1186/s12960-016-0107-x

29. Kenya Ministry of Health. Scope of practice for ophthalmic workers. 2022. Available: https://www.health.go.ke/

30. International Agency for Prevention and Blindness. Core competency for the eye health workforce in the WHO African region. 2016. Available: https://www.iapb.org/wp-content/uploads/Core-Competencies-for-Eye-HWF-in-WHO-AFRO-Region.pdf

31. Morjaria P, Ramson P, Gichangi M. Services for refractive error in Kenya. J Ophthalmol East Cent South Africa. 2013. Available: https://www.researchgate.net/publication/279926992

32. Morjaria P, Minto H, Ramson P, Gichangi M, Naidoo K, Gilbert C. Services for refractive error in Kenya : extent to which human resources and equipment are meeting VISION 2020 targets. J Ophthalmol East Cent South Africa. 2013;17: 44–49. Available: http://coecsa.org/ojs-2.4.2/index.php/JOECSA/article/view/78

33. Patel I, West SK. Presbyopia : prevalence , impact , and interventions. Community Eye Heal J. 2007;20: 51–52.

34. Eccles MP, Weijer C, Mittman B. Requirements for ethics committee review for studies submitted to Implementation Science. Implement Sci. 2011;6: 32. doi:10.1186/1748-5908-6-32

35. Leong SL, Teoh SL, Fun WH, Lee SWH. Task shifting in primary care to tackle healthcare worker shortages: An umbrella review. Eur J Gen Pract. 2021;27: 198–210. doi:10.1080/13814788.2021.1954616

36. Palmer J, Chinanayi F, Gilbert A, Pillay D, Fox S, Jaggernath J, et al. Mapping human resources for eye health in 21 countries of sub-Saharan Africa: current progress towards

VISION 2020. Hum Resour Health. 2014;12. doi:10.1186/1478-4491-12-44

37. Ramke J, Zwi AB, Silva JC, Mwangi N, Rono H, Gichangi M, et al. Evidence for national universal eye health plans. Bull World Health Organ. 2018;96: 695–704. doi:10.2471/BLT.18.213686

38. KK Optic. What is the Cost of Prescriptive Eyeglasses in Kenya? - KK Opticians. 2022. Available: https://kkopticals.co.ke/

39. Lapaire Glasses. Quality & Affordable Eyeglasses in Africa | Lapaire Glasses. 2018 [cited 7 May 2023]. Available: https://lapaire.africa/

40. Patel I, West SK. Presbyopia: prevalence, impact, and interventions. Community eye Heal. 2007;20: 40–41.

41. University of Nairobi. Department of Ophthalmology fee and funding. 2023 [cited 7 May 2023]. Available: https://opthalmology.uonbi.ac.ke/programs-content-type/master-medicine-ophthalmology

42. Masinde Muliro University. Bachelor of Optometry fee structure. 2019 p. 597360. Available: https://www.mmust.ac.ke/index.php/student-fee-structures

43. Kenya Medical Training College. Kenya Medical Training College | Training For Better Health. 2022. Available: https://kmtc.ac.ke/

44. Kenya National Bureau of Statistics. 2019 Kenya Population and Housing Census. 2019. Available: https://www.knbs.or.ke/2019-kenya-population-and-housing-census-reports/

45. International Agency for Prevention and Blindness. Strategy for the Elimination of Vision Impairment from Uncorrected Refractive Error. 2022. Available: https://www.iapb.org/learn/resources/strategy-for-the-elimination-of-vision-impairment-from-uncorrected-refractive-error/

46. Kenya Ministry of Health. National Strategic Plan For Eye Health and Blindness Prevention2012 to 2018. 2011.

47. Naidoo KS, Fricke TR, Frick KD, Jong M, Naduvilath TJ, Resnikoff S, et al. Potential Lost Productivity Resulting from the Global Burden of Myopia: Systematic Review, Meta-analysis, and Modeling. Ophthalmology. 2019;126: 338–346. doi:10.1016/j.ophtha.2018.10.029

48. Courtright P, Mathenge W, Kello AB, Cook C, Kalua K, Lewallen S. Setting targets for human resources for eye health in sub-Saharan Africa: What evidence should be used? Human Resources for Health. BioMed Central Ltd.; 2016. doi:10.1186/s12960-016-0107-x

49. Palmer JJ, Chinanayi F, Gilbert A, Pillay D, Fox S, Jaggernath J, et al. Mapping human resources for eye health in 21 countries of sub-Saharan Africa: Current progress towards VISION 2020. Hum Resour Health. 2014;12. doi:10.1186/1478-4491-12-44

50. Yorston D, Buchan J. Investment in human resources improves eye health for all. Community Eye Heal J. 2018;35: 37–39.

51. Khan AA, Khan NU, Bile KM, Awan H. Creating synergies for health systems strengthening through partnerships in Pakistan--a case study of the national eye health programme. East Mediterr Heal J. 2010;16 Suppl: S61-8.

52. Marques AP, Ramke J, Cairns J, Butt T, Zhang JH, Jones I. The economics of vision impairment and its leading causes: A systematic review. Clin Med (Northfield Il). 2022;46: 101354. doi:https://doi.org/10.1016/j.eclinm.2022.101354

53. World Bank. Macro Poverty Outlook for Kenya. 2022 pp. 1–2. Available: https://www.worldbank.org/en/publication/macro-poverty-outlook

54. World Health Organization. World report on vision. 2019. doi:http://apps.who.int/iris.
55. Kenya Laws. The constitution of Kenya, 2010. 2010. Available: http://kenyalaw.org/kl/index.php?id=398
56. Luke B, Chu V. Social enterprise versus social entrepreneurship : An examination of the ' why ' and ' how ' in pursuing social change. Int Small Bus J. 2013;31: 765–766. doi:10.1177/0266242612462598
57. Nyawira L, Tsofa B, Musiega A, Munywoki J, Njuguna RG, Hanson K, et al. Management of human resources for health: implications for health systems efficiency in Kenya. BMC Health Serv Res. 2022;22: 1046. doi:10.1186/s12913-022-08432-1
58. Haleem A, Javaid M, Singh RP, Suman R. Telemedicine for Healthcare : Capabilities , features , barriers , and applications Telemedicine for healthcare : Capabilities , features , barriers , and applications. Sensors Int. 2021;2: 100117. doi:10.1016/j.sintl.2021.100117
59. Kim T, Zuckerman JE. Realizing the potential of telemedicine in global health. J Glob Health. 2019;9. doi:10.7189/jogh.09.020307
60. Chia MA, Turner AW. Benefits of Integrating Telemedicine and Artificial Intelligence Into Outreach Eye Care: Stepwise Approach and Future Directions. Front Med. 2022;9: 1–6. doi:10.3389/fmed.2022.835804
61. Randhawa H, Morettin C, McLeod H, Wyles E, Sanghera N, Kattouf V, et al. The validity of spectacle prescriptions via tele-optometric comprehensive eye examinations; a pilot study. Invest Ophthalmol Vis Sci. 2020;61: 1604.
62. Ulldemolins AR, Lansingh VC, Valencia LG, Carter MJ, Eckert KA. Review Article Social inequalities in blindness and visual impairment : A review of social determinants. 2011. doi:10.4103/0301-4738.100529

63. Zhang JH, Ramke J, Jan C, Bascaran C, Mwangi N, Furtado JM, et al. Review Advancing the Sustainable Development Goals through improving eye health : a scoping review. Lancet Planet Heal. 2022; 270–280. doi:10.1016/S2542-5196(21)00351-X

Dear Dr. Ogbomo (Academic Editor – Plos One)

Detailed below are the revisions made to the article titled '**Estimation of the lost productivity to the GDP and the national cost of correcting visual impairment from refractive error in Kenya** (Manuscript. No. PONE-D-23-29248)'. The changes were made based on the comments/suggestions of **Reviewer #1, Reviewer #2, Reviewer #3 and the Academic Editor.** All changes have been tracked on the revised manuscript. The authors' responses to queries received are formatted through word track changes.

ACADEMIC EDITOR:

Before acceptance please ensure the following has been addressed:

This is noted

Address the queries of each review as clearly as possible.

This is noted and has been addressed across the document

Undertake extensive English Language edit of the entire manuscript

Editing has been undertaken across the document

Reviewer #1: Thank you for your submission. The paper is relevant and highlights the importance of accessibility to vision services and optical corrective devices and the inequality that exists across the world. However, review of the paper with a recommendation of major review, is required.

Please see the main points below and detail comments in the manuscript:

As English is not your first language the paper would benefit form a proof-reading by a native speaker or a proof-reading service. Some sentences are formulated in an unclear fashion or are incomplete.

This is noted and has been addressed across the document as suggested in the reviewed manuscript

Try to avoid terms like cadres or refraction points. Alternatives have been provided in comments in the manuscript.

This is noted. Changes have been made across the document as suggested

References should be reviewed and formatted in accordance to the publishers guidance. Some of them are incomplete, some have invalid links attached. Organisations such as WHO should be written in full in reference list.

This has been addressed as suggested

In body of the manuscript, when referring to organisations, e.g. WHO and others, first mention name in full, then abbreviate.

Thanks. This has been revised accordingly across the document

Some of the estimations and calculations could be explained clearer.

This is noted and has been addressed across the document. Line 80-264

The various ratios and recommendations should be referenced.

Yes the recommendations have been referenced. Line 153-156, 159-162, 237-238, 262-263

Think about how you would integrate telemedicine in your vision centres as in optometry, it has a limited role and can be used for triaging the patient, obtaining basic history 7 symptoms and for follow ups with the patient. This also would require a infrastructure (phones, internet for video calls with patient and other colleagues). If you are referring to telemedicine hubs and for eyecare professionals to communicate between themselves in regards to a patient/patient presentation or course of treatment or management, please specify that. 144-146

This is noted. “If you are referring to telemedicine hubs and for eyecare professionals to communicate between themselves in regards to a patient/patient presentation or course of treatment or management”. Yes this is what we referred to. Detailed on page 148-150

Reviewer #2: I find the topic interesting and important to the eye care services in Kenya. From my point of view, the paper needs to be revised and improved.

This is noted and has been addressed as suggested

First, the authors don´t provide a definition for Refractive error. That is a critical point since that will have an impact on the age group that should be considered to estimate productivity losses.

The definition has been provided in line 54-55

It is unclear why they decided to use two age groups 16-50 and 16-60.

This is noted. We would like to clarify that the initial estimates was based on the first study on global estimate of the cost required to address URE and the lost productivity to the GDP. However, with the advancement in the approaches used, we have made the calculations using the latest development (A Simple Method for Estimating the Economic Cost of Productivity Loss Due to Blindness and Moderate to Severe Visual Impairment by Eckert and colleagues). Therefore, this study adopts the 16-60 years only. Details are provided in line 80-92.

Why should people above the age of 50 y.o be considered unproductive if the retirement age in the country is 60 y.o.

The unproductive age in the study has been rectified as individuals below 16 years and those above 60 years. Line 91

They also don´t provide a definition for labour force participation and employment rate which I believe would be useful to do.

This is noted. The definitions have been provided in line 108-113

Second, they use disability weights to measure productivity losses without providing a good rational to do it. Disability weights and productivity losses should not be seen as substitutes. They measure different aspects of life. Disability weights represent the magnitude of health loss while productivity losses represent "the production loss due to illness, disability and death of productive persons, both paid and unpaid".

This is noted. Based on the current estimate approach, the disability weights were not used instead the minimum wage, the affected population and reduced earnings was used as proposed in a study by Eckert and colleagues: A Simple Method for Estimating the Economic Cost of Productivity Loss Due to Blindness and Moderate to Severe Visual Impairment. Line 80-92 provides the details

Third, it is not clear why are they using the all population (either 16-50 or 16-60) and not just the population who have refractive error (which will vary with the prevalence rate of refractive error in Kenya).

This is noted. The current estimate is based on the 3.5 million Kenyans with URE. Line 86-92 details

Forth, I think there is a mistake with the reference of the disability weights used in the paper. Salomon´s et al (ref 6) report disability weights of 0.003 for mild visual impairment, 0.031 for moderate visual impairment and 0.184 for severe visual impairment. The disability weights used in the paper are proposed by WHO. Authors should explain why they did not use the latest disability weights figures.

This is noted. The disability weight approach has been eliminated with adoption of the latest formula for estimating the productivity loss. Therefore there is shift from the initial formula to the latest. Details are provided in the methodology section line 80-211

Fifth, there is something wrong with table 1 and table 2 (and with the way productivity losses are estimated). The product between number of people (Column 1 population aged 16-50 years) and disability weights (Column 2 population aged 16-50 years) can not directly produce a value expressed in USD.

We agree and based on the current formula, we have made changes in line 213-226

Finally, the national cost estimation and cost benefit analysis are not well describe and needs to be revised.

This has been revised as proposed in line 287-305 and line 227-272 in the revised document

The reference list doesn´t follow common reference styles.

This is noted and has been addressed as instructed

Reviewer #3: The study is interesting and makes significant contribution to the economics of uncorrected refractive errors in Kenya

The study will require language editing

This is noted and we have edited across the document

Clarify how the average income of the normal sighted caregiver aged 16 – 60 years was calculated

The minimum wage in Kenya is approximately US$ 101.505. Given that approximately 7.5% of the individuals with URE in Kenya have severe VI, we computed the annual income with an assumption that they constitute the LFPR. Details are provided on line 219-226

Line 130 – 135. Why was the assumption of time spent in doing refraction leave out the cadre of optometrists?

Optometrists are core and in this study we consider them as functional clinical refractionists. The WHO outline that optometrists are well placed to address URE. Line 230-232 provides the estimates.

Line 135 – 139. Are optometrists classified under the clinical refractionist cadre? Please explain to provide clarity. Yes, the details are in line 166-167

Referencing style should follow journal’s guidelines.

This is noted and has been addressed under references sub-heading
