# Supplementary material for: Estimation of the lost productivity to the GDP and the national cost of correcting visual impairment from refractive error in Kenya
Source: PLoS One. 2024 Mar 25;19(3):e0300799. doi: 10.1371/journal.pone.0300799 (PMC10962815; doi:10.1371/journal.pone.0300799)
Supplement: Attachment — Submitted filename: Responses to Reviewers.docx [file pone.0300799.s003.docx]

Dear Dr. Ogbomo (Academic Editor – Plos One)

Detailed below are the revisions made to the article titled ‘**Estimation of the lost productivity to the GDP and the national cost of correcting visual impairment from refractive error in Kenya** (Manuscript. No. PONE-D-23-29248)’. The changes were made based on the comments/suggestions of **Reviewer #1, Reviewer #2, Reviewer #3 and the Academic Editor.** All changes have been tracked on the revised manuscript. The authors’ responses to queries received are formatted through word track changes.

ACADEMIC EDITOR:

Before acceptance please ensure the following has been addressed:

This is noted

Address the queries of each review as clearly as possible.

This is noted and has been addressed across the document

Undertake extensive English Language edit of the entire manuscript

Editing has been undertaken across the document

Reviewer #1: Thank you for your submission. The paper is relevant and highlights the importance of accessibility to vision services and optical corrective devices and the inequality that exists across the world. However, review of the paper with a recommendation of major review, is required.

Please see the main points below and detail comments in the manuscript:

As English is not your first language the paper would benefit form a proof-reading by a native speaker or a proof-reading service. Some sentences are formulated in an unclear fashion or are incomplete.

This is noted and has been addressed across the document as suggested in the reviewed manuscript

Try to avoid terms like cadres or refraction points. Alternatives have been provided in comments in the manuscript.

This is noted. Changes have been made across the document as suggested

References should be reviewed and formatted in accordance to the publishers guidance. Some of them are incomplete, some have invalid links attached. Organisations such as WHO should be written in full in reference list.

This has been addressed as suggested

In body of the manuscript, when referring to organisations, e.g. WHO and others, first mention name in full, then abbreviate.

Thanks. This has been revised accordingly across the document

Some of the estimations and calculations could be explained clearer.

This is noted and has been addressed across the document. Line 80-264

The various ratios and recommendations should be referenced.

Yes the recommendations have been referenced. Line 153-156, 159-162, 237-238, 262-263

Think about how you would integrate telemedicine in your vision centres as in optometry, it has a limited role and can be used for triaging the patient, obtaining basic history 7 symptoms and for follow ups with the patient. This also would require a infrastructure (phones, internet for video calls with patient and other colleagues). If you are referring to telemedicine hubs and for eyecare professionals to communicate between themselves in regards to a patient/patient presentation or course of treatment or management, please specify that. 144-146

This is noted. “If you are referring to telemedicine hubs and for eyecare professionals to communicate between themselves in regards to a patient/patient presentation or course of treatment or management”. Yes this is what we referred to. Detailed on page 148-150

Reviewer #2: I find the topic interesting and important to the eye care services in Kenya. From my point of view, the paper needs to be revised and improved.

This is noted and has been addressed as suggested

First, the authors don´t provide a definition for Refractive error. That is a critical point since that will have an impact on the age group that should be considered to estimate productivity losses.

The definition has been provided in line 54-55

It is unclear why they decided to use two age groups 16-50 and 16-60.

This is noted. We would like to clarify that the initial estimates was based on the first study on global estimate of the cost required to address URE and the lost productivity to the GDP. However, with the advancement in the approaches used, we have made the calculations using the latest development (A Simple Method for Estimating the Economic Cost of Productivity Loss Due to Blindness and Moderate to Severe Visual Impairment by Eckert and colleagues). Therefore, this study adopts the 16-60 years only. Details are provided in line 80-92.

Why should people above the age of 50 y.o be considered unproductive if the retirement age in the country is 60 y.o.

The unproductive age in the study has been rectified as individuals below 16 years and those above 60 years. Line 91

They also don´t provide a definition for labour force participation and employment rate which I believe would be useful to do.

This is noted. The definitions have been provided in line 108-113

Second, they use disability weights to measure productivity losses without providing a good rational to do it. Disability weights and productivity losses should not be seen as substitutes. They measure different aspects of life. Disability weights represent the magnitude of health loss while productivity losses represent “the production loss due to illness, disability and death of productive persons, both paid and unpaid”.

This is noted. Based on the current estimate approach, the disability weights were not used instead the minimum wage, the affected population and reduced earnings was used as proposed in a study by Eckert and colleagues: A Simple Method for Estimating the Economic Cost of Productivity Loss Due to Blindness and Moderate to Severe Visual Impairment. Line 80-92 provides the details

Third, it is not clear why are they using the all population (either 16-50 or 16-60) and not just the population who have refractive error (which will vary with the prevalence rate of refractive error in Kenya).

This is noted. The current estimate is based on the 3.5 million Kenyans with URE. Line 86-92 details

Forth, I think there is a mistake with the reference of the disability weights used in the paper. Salomon´s et al (ref 6) report disability weights of 0.003 for mild visual impairment, 0.031 for moderate visual impairment and 0.184 for severe visual impairment. The disability weights used in the paper are proposed by WHO. Authors should explain why they did not use the latest disability weights figures.

This is noted. The disability weight approach has been eliminated with adoption of the latest formula for estimating the productivity loss. Therefore there is shift from the initial formula to the latest. Details are provided in the methodology section line 80-211

Fifth, there is something wrong with table 1 and table 2 (and with the way productivity losses are estimated). The product between number of people (Column 1 population aged 16-50 years) and disability weights (Column 2 population aged 16-50 years) can not directly produce a value expressed in USD.

We agree and based on the current formula, we have made changes in line 213-226

Finally, the national cost estimation and cost benefit analysis are not well describe and needs to be revised.

This has been revised as proposed in line 287-305 and line 227-272 in the revised document

The reference list doesn´t follow common reference styles.

This is noted and has been addressed as instructed

Reviewer #3: The study is interesting and makes significant contribution to the economics of uncorrected refractive errors in Kenya

The study will require language editing

This is noted and we have edited across the document

Clarify how the average income of the normal sighted caregiver aged 16 – 60 years was calculated

The minimum wage in Kenya is approximately US$ 101.505. Given that approximately 7.5% of the individuals with URE in Kenya have severe VI, we computed the annual income with an assumption that they constitute the LFPR. Details are provided on line 219-226

Line 130 – 135. Why was the assumption of time spent in doing refraction leave out the cadre of optometrists?

Optometrists are core and in this study we consider them as functional clinical refractionists. The WHO outline that optometrists are well placed to address URE. Line 230-232 provides the estimates.

Line 135 – 139. Are optometrists classified under the clinical refractionist cadre? Please explain to provide clarity. Yes, the details are in line 166-167

Referencing style should follow journal’s guidelines.

This is noted and has been addressed under references sub-heading
