# Supplementary material for: Estimation of the lost productivity to the GDP and the national cost of correcting visual impairment from refractive error in Kenya
Source: PLoS One. 2024 Mar 25;19(3):e0300799. doi: 10.1371/journal.pone.0300799 (PMC10962815; doi:10.1371/journal.pone.0300799)
Supplement: Attachment — Submitted filename: Responses to Reviewers.docx [file pone.0300799.s005.docx]

Dear Dr. Ogbomo (Academic Editor – Plos One)

Detailed below are the revisions made to the article titled ‘**Estimation of the lost productivity to the GDP and the national cost of correcting visual impairment from refractive error in Kenya** (Manuscript. No. PONE-D-23-29248_R1)’. The changes were made based on the comments/suggestions of **Reviewer #1, Reviewer #3 and the Academic Editor.** All changes have been tracked In the revised manuscript.

ACADEMIC EDITOR:

Please address the concern of the having the recommendation in the methods section of the manuscript. The recommendation should typically be in the discussion.

**This is noted and has been transferred to the discussion as proposed.**

Additional Editor Comments:

As note by Reviewer 1, the recommendations noted in the methods section should typically be made in the discussion section of the manuscript if the recommendations are from the current paper. Please address these and a few other comments suggested.

**This is noted and has been corrected in the revised version line 383-386. The other comments by reviewer 1 have been addressed across the document.**

Review Comments to the Author

Reviewer #1: The manuscript has been improved significantly - well one. There are still some mistakes in the use of language & some things like VI & URE or how to correct RE should be explained/defined

**Thank you. The suggestion has been integrated in line 55-59 and 62-64 as suggested.**

Also, look at methodology/results - study recommendations should be included in discussion not results or methodology. You can use suggestions or estimations for calculations (see comments in the text). Therefore, minor revisions are still needed.

**This is noted and such recommendations have been transferred to the discussion section line 383-386.**

Reviewer #3: The authors have addressed the all comments i raised satisfactorily. This has improved the quality of the paper

**Thank you**
